# Revisiting port sustainability as a foundation for the implementation of the United Nations Sustainable Development Goals (UN SDGs)

**DOI:** 10.1186/s41072-021-00101-6

**Published:** 2021-11-08

**Authors:** Anas S. Alamoush, Fabio Ballini, Aykut I. Ölçer

**Affiliations:** World Maritime University, P.O. Box 500, 201 24 Malmö, Sweden

**Keywords:** Ports, Sustainability, Actions and measures, UN SDGs, Implementation, Triple bottom lines, TBLs

## Abstract

Port sustainability studies are increasing rapidly and are skewed toward environmental aspects, while at the same time their results are fragmented, making it difficult to collectively assess conclusions. This study, therefore, aims at building a framework which categorises port actions, measures, and implementation schemes (policy tools to improve the uptake), utilising the critical literature review method. Additionally, linkage of port sustainability to the United Nations’ Sustainable Development Goals (UN SDGs) is highlighted. Port sustainability includes internal (port side) and external (ships and land transport) actions and measures. The study results form 16 homogeneous and interconnected sustainability categories, including a non-exhaustive list of operationalising measures, encompassing the three dimensions of sustainability (environment, economy and society) while implementation schemes are divided into four groups. Considering that ports are under scrutiny and perceiving growing pressure to improve their sustainable pathways, for example by addressing climate change and energy consumption, the identified ports’ sustainability actions and measures, including the linkage with the UN SDGs, are overarching and multidimensional and seen as a step in achieving far-reaching sustainable implementation. The study’s practical implications guide port policymakers and industry practitioners to go beyond the low hanging fruit (customary practices), and enable them to take reliable decisions for broader sustainability implementation. Additionally, the identified research implications stimulate further academic discussions.

## Introduction

Ports play a central role in countries’ economic growth: they are essential to the wellbeing of humankind including the provision of direct and indirect employment (Roh et al. 2016). Ports act as a social caretaker for employees and communities, enhancing and supporting socioeconomic priorities. In Europe, 2200 port operators employ more than 110,000 workers who are engaged in loading and unloading ships and in port-based services such as warehousing and logistics (Van Hooydonk 2014). On the other hand, ports are inevitable nodes in maritime supply chains (Asgari et al. 2015; Poulsen et al. 2018; Notteboom et al. 2020).

Considered as gateways to international trade, there exist thousands of seaports handling seaborne trade. As of 2018, some 98,140 ships carried 11 billion tons of seaborne trade, which is around 80% and 60–70% of world trade volume and value, respectively (UNCTAD 2019a). Only in 2019, ships of 100 gross tons and above made 4,362,737 port calls.^1^ Even in the worst shocks, particularly the recent COVID-19 pandemic, ports and shipping were at the global transport forefront, maintaining continuous delivery of the world’s medical supplies, food, energy, and raw materials, as well as manufactured goods and components (UNCTAD 2020a). However, considering the magnitude of port activities, ports as nodes in the global supply chains always generate social and environmental externalities (Darbra et al. 2004; Dinwoodie et al. 2012) vis a vis economic growth. In general, ports generate environmental impacts through their various functions linked to cargo handling, connectivity to maritime and land transport networks, industrial and semi-industrial activities, logistics and distribution activities, and energy production and distribution (Notteboom et al. 2020). Such external impacts (*externalities*), both from port expansion and operations, and from the activities of shipping and land transport, have severe impacts on the environment (Darbra et al. 2004; Peris-Mora et al. 2005; Dinwoodie et al. 2012; Acciaro et al. 2014). Ports’ impacts extend to oceans and seas, and worsen marine ecosystems (Darbra et al. 2009), even though oceans are pivotal to global and national economies by providing food, jobs and recreational activities.

The concepts of minimising port environmental externalities, including steering economic growth, and addressing societal needs, are all included in so-called port sustainability (Cheon 2017; Cheon et al. 2017; Laxe et al. 2017). In other words, sustainability encompasses the triple bottom lines (TBLs), i.e., economic, environmental and social dimensions (Elkington 1998; Gimenez et al. 2012). This also applies for the port sustainability. Akin to the importance of port sustainability in internal operations is that ports extend sustainability externally to landside transport and shipping at the sea side (Roh et al. 2016; Laxe et al. 2017; Oh et al. 2018). It has been demonstrated that ports have roles to play in greening maritime transport and supply chains (Asgari et al. 2015; Notteboom et al. 2020) and in accelerating environmental upgrading (Poulsen et al. 2018). For example, ports facilitate shipping GHG emission reduction (ITF/OECD 2018; Alamoush et al. 2020). By doing so, ports move past the customary environmental initiatives (low hanging fruit) into a more holistic sustainability that plans for TBLs internally and externally (I2S2 2013; Puig et al. 2014; Acciaro et al. 2014).

The quest for port sustainability has accelerated due to increased scrutiny of ports and pressure to take actions and decrease externalities through sustainable and cleaner operations (UNCTAD 2019a). Such pressure motivates and stimulates ports not to merely focus on economic generation, but also to include resilient sustainable strategies (Lu et al. 2016a). Put differently, ports are required to balance commercial and economic growth against environmental and social sustainability (Stein and Acciaro 2020), and thus to achieve competitive advantage and boost service quality. Pressure on ports is driven by, inter alia, local and international regulations (Lam and Notteboom 2014), local communities and non-governmental organisations (NGOs) (Lee et al. 2015; MTCC Pacific 2017; IMO 2018a), corporate social responsibility (CSR) (Woo et al. 2018), energy efficiency economic benefits (Acciaro and Wilmsmeier 2015; Wilmsmeier and Spengler 2016), shippers,^2^ consignees, cargo owners and carriers (Poulsen et al. 2018; Jasmi and Fernando 2018), environmental awareness and pursuit of a green port image (Notteboom et al. 2020). Overall, ports that feature a high pro-environmental attitude improve economic efficiency (Castellano et al. 2020).

Revisiting research on port sustainability can be justified because there are existing research and practice issues (gaps). *From an academic perspective*, the extant literature provides an array of measures to decrease port externalities, either as a group of measures in the green port concept (e.g. Lirn et al. 2013; Chiu et al. 2014; Lam and Notteboom 2014; PIANC 2014; Davarzani et al. 2016; Bergqvist and Monios 2019), or in the sustainable port concept (e.g., (I2S2 2013; Asgari et al. 2015; Bjerkan and Seter 2019; Lim et al. 2019). Single port sustainability measures have also been studied, such as air quality improvement (Corbett et al. 2007), energy efficiency (Iris and Lam 2019), greenhouse gas (GHG) emission reduction (Alamoush et al. 2020), renewable energy (PIANC 2019), alternative fuel (Zhong et al. 2019), electrification of cargo handling equipment (CHE) (Yang and Chang 2013), noise reduction (Enguix et al. 2019), and climate change adaptation (Wilmsmeier 2020). In view of the above studies, the first gap that can be gleaned is that sustainability actions and measures are addressed mainly within the environmental dimensions, e.g., (Darbra et al. 2009; Lirn et al. 2013; Lam and Notteboom 2014; Acciaro et al. 2014; Davarzani et al. 2016). Ports’ economic and social dimensions are not well addressed in the literature, though few studies addressed all the three sustainability dimensions (TBLs), e.g., (Shiau and Chuang 2013; Sislian et al. 2016; Roh et al. 2016; Laxe et al. 2017; Oh et al. 2018; Lim et al. 2019). Secondly, port sustainability within internal operations is the centre of attention in many studies while other relevant areas (e.g., land transport, and shipping) are not widely addressed (Roh et al. 2016; Lim et al. 2019; Castellano et al. 2020; Hossain et al. 2020). Thirdly, the focus remains on port sustainability assessment indicators, and, if addressed, the measures and actions are fragmented and available in heterogeneous formats, i.e., not totally aggregated as a one tool. Addressing these attributes separately could lead to partial analysis and incomplete conclusion (Castellano et al. 2020). Fourthly, how to implement ports sustainability actions and measures—drive and increase the uptake—is not broadly dealt with. Last but not least, the United Nations Sustainable Development Goals (UN SDGs)—2030 Agenda—were introduced in 2015 as a solution to wide-ranging global sustainability (United Nations 2015). The UN SDGs aim at “eradicating poverty in all its forms and dimensions, combating inequality within and among countries, preserving the planet, creating sustained, inclusive and sustainable economic growth and fostering social inclusion” (United Nations 2015). Ports functions are various, enabling them to have a broader role in UN SDGs implementation and promotion (WPSP 2020). Zooming out to a global perspective, it could be argued that port sustainability actions contribute to sustainability in general and more specifically to achieving the UN SDGs due to some commonality in addressing the TBLs. However, studies rarely shed light on this important association.

From a *practice* point of view, while some of the above-mentioned port sustainability actions and measures are mainly implemented by front-runner ports in Europe, North America, and a handful of ports in Asia (Poulsen et al. 2018; Bjerkan and Seter 2019; Alamoush et al. 2020; Hossain et al. 2020), some others are only proposed to set ports on the rightful sustainability track. It could be argued that issues in ports’ economy, regulations execution, institutional governance, organisational and information barriers, business models, and geography, among others, may have decelerated implementation (Alamoush et al. 2021b). In addition, the COVID-19 pandemic negatively influenced ports and shipping operations, and sustainable projects and performance (IMO 2020a; Notteboom and Pallis 2020a, 2020b; Alamoush et al. 2021c), and slowed the progress of implementation of the 17 United Nations Sustainable Development Goals (UN SDGs) (IMO 2020b). Therefore, the study of how ports improve implementation of sustainability actions is deemed necessary. Secondly, while seaborne trade growth decreased in 2020, due to the Pandemic (UNCTAD 2020b), it was projected to bounce back relatively firmly in 2021 signalling further growth (around 4%) to above the 2019 level (Clarksons Research 2020a). Considering this anticipated increase, it should be borne in mind that typically, while ports handle seaborne trade (cargo throughput), ecological and environmental issues amplify, and demand for energy increases. As a consequence, the best way forward is to maintain a sustainable performance during such recovery, i.e., defending environmental, social, and economic growth (Clarksons Research 2020b; UNCTAD 2020b, 2020c, 2020a). Hence, illustrating port sustainability from holistic approach and aggregating all actions and measures in a one-stop shop (tool) is advantageous for port practitioners that intend to integrate sustainability in port operations.

Given the pressure on ports to maintain sustainable performance including having a broader role in sustainable development, and given the aforementioned academic and practice gaps, this study aims at building a framework that aggregates the ports’ overall sustainability actions and measures, and identifies the implementation schemes that put into action the TBLs of sustainability in the landside and sea side (i.e. internally and externally). While at the same time this study aims at identifying ports’ role in the implementation of UN SDGs. Utilising a critical literature review method, the study is guided by three questions: RQ1: What are the categories of ports’ actions and measures to improve overall port sustainability internally in the port side and externally in the sea side (shipping), and in land transport (trucks)?; RQ2: How port sustainability actions and measures can be implemented by public and port authorities to drive the uptake of actions and measures (implementation schemes/tools)?; and RQ3: What is the linkage between port sustainability actions and measures and the United Nations Sustainable Development Goals (UN SDGs)?

Although there exist various reviews that address port sustainability (e.g., Asgari et al. 2015; Davarzani et al. 2016; Bjerkan and Seter 2019; Lim et al. 2019)), this study builds on these previous reviews to revisit port sustainability and address current gaps with a focus of linking port sustainability with UN SDGs. In so doing, this study contributes to academic research, and policy and practice. Academically, the study integrates developments in the field of port sustainability by: building categorisation of findings (actions and measures), developing a conceptual framework that posits new relationships and perspectives on the topic, and suggesting an agenda of future research that serves as a ground for further investigation of port sustainable actions and focuses on measures to reduce ports’ externalities. Additionally, the study contributes to the global sustainable development implementation. On the practice side, the result of this study is considered to be a comprehensive tool of wide-ranging sustainability actions and measures which informs port practitioners and policy makers and assists them to take reliable decisions. It thus enables them to gauge their advancement or decline in sustainability, and to see how to improve implementation. As far as authors are aware, this is the first study that builds up holistic port sustainability measures and actions with such a large scope and different dimensions while addressing the UN SDGs concept.

While the introduction has provided a background for this study and explained its relevance, the next Section covers “Materials and methods”, “Literature review: building a port sustainability framework” Section covers the building of the port sustainability framework (literature review), “Internal and external ports’ sustainability actions and measures and the association with SDGs” Section covers internal and external port sustainability actions and measures, “Linkage of port sustainability actions and measures to the UN SDGs” Section covers the actions’ and measures’ linkage to the UN SDGs, and “Discussions and conclusions” Section contains the discussion and conclusions.

## Materials and methods

This study uses the critical literature review method to answer the research questions. The main goal of this research is to categorise sustainability actions in ports along with essential measures that fulfil and implement these actions. This facilitates the exploration of the linkage between port sustainability and the UN SDGs.

Since there are no standard methods for developing categories (taxonomies) of actions and measures; previous studies followed different approaches (e.g., an exploratory review of green port measures (Lam and Notteboom 2014), a systematic review of technologies and tools of port sustainability (Bjerkan and Seter 2019), a systematic literature review of ports’ GHG emission reduction measures (Alamoush et al. 2020, 2021b), and qualitative thematic analysis to build topologies of barriers to the female gender in shipping (Kim et al. 2019), among others). In this research we utilise the critical literature review based on guidelines in (Grant and Booth 2009; MacInnis 2011; Snyder 2019; Jaakkola 2020). A range of studies utilised the critical literature review approach, e.g., building typology of circular economy discourses (Friant et al. 2020), and determinants of online information search (Kulviwat et al. 2004).

The critical literature review approach integrates the literature with the aim of assessing, critiquing and synthesizing the literature on a particular concept so that new frameworks and perspectives arise (Snyder 2019). The emphasis in the critical literature review is on the innovative collection of data from sufficient established research in the field, while not covering every study therein. This leads to a combination of different perspectives and insights (Torraco 2005). For mature topics (i.e., port sustainability), critical literature review revisits the knowledge base, analytically reviews and potentially revises concepts and thus expands the theoretical foundation of a continuously developing topic (Snyder 2019). While critical literature review could be described as a weak tool due to the subjectivity in selection of included studies, systematic literature review, on the contrary, avoids such bias by having criteria for inclusion and exclusion of studies (Petticrew and Roberts 2008; Denyer and Tranfield 2009). However, a systematic review is more commonly based on academic peer-reviewed studies, and thus excludes grey literature (e.g., book chapters, proceedings, and technical reports), which are allowed in critical reviews, by searching Google Scholar for example. Most of reviews in this field are systematic, and variation of methods is seen necessary to generate new insights and avoid strict systematic criteria.

**Fig. 1:**
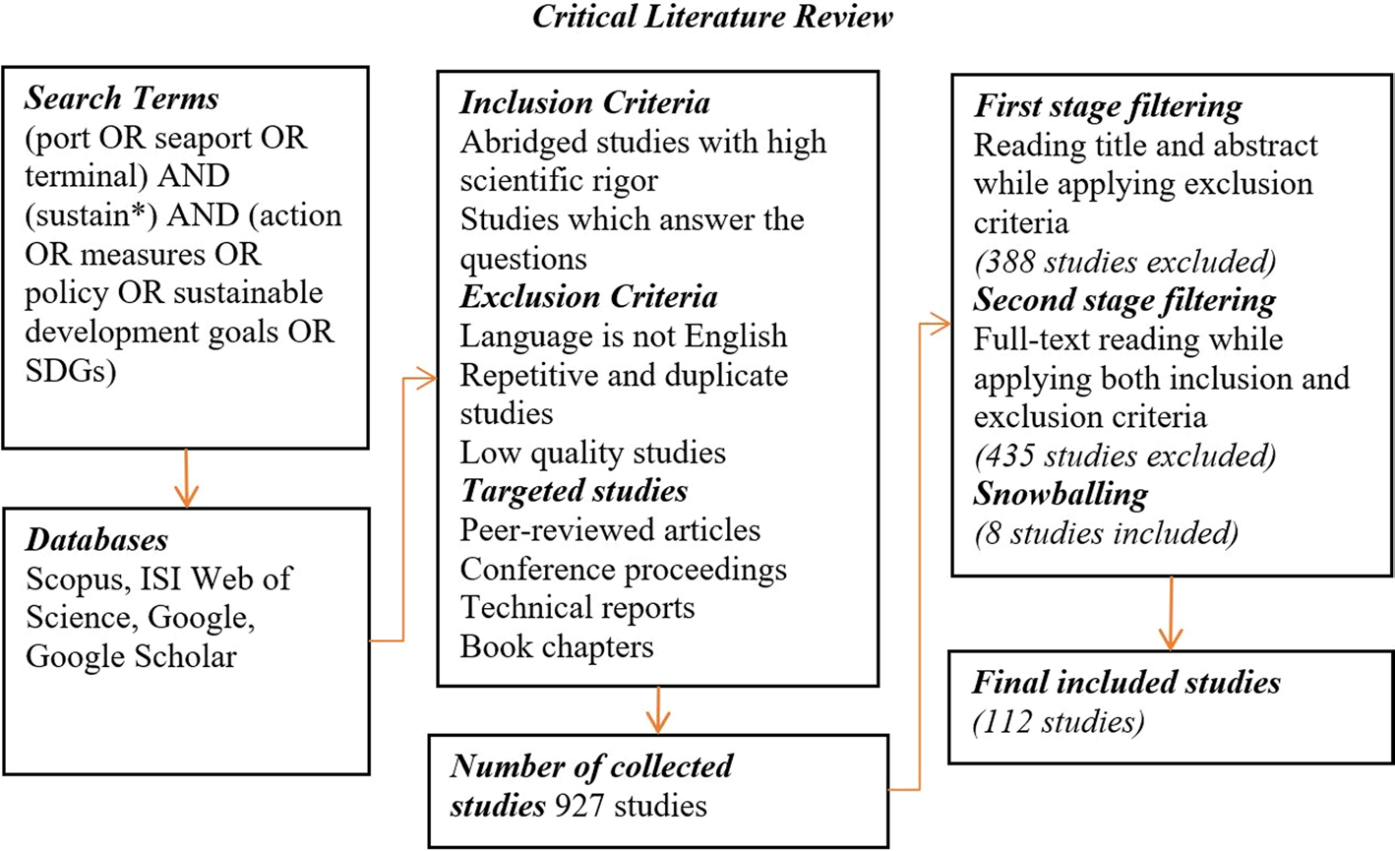
Flowchart of the critical literature review steps including inclusion and exclusion criteria and filtering stages. *Note* Scientific rigor in the inclusion criteria is determined based on application of proper scientific methods which guarantee unbiased and well-established design, methodology, analysis, interpretation and reporting

While answering the study’s questions entails establishing themes and categories that bring about broader perspectives, i.e., not investigating in depth specific studies; the critical literature review method used in this study is seen as suitable, and so helps avoid integrating repetitive results. Thus, academic peer-reviewed studies are included, in addition to grey literature such as European and North American technical reports. The basics of systematic review search have been applied to improve the credibility of search and studies collection. Figure  1 illustrates the review steps together with inclusion and exclusion criteria and filtering stages.

As can be seen in Fig. 1, studies were collected by searching various data bases in March 2021: Scopus, ISI Web of Science in addition to the utilisation of Google and Google Scholar to retrieve technical reports. The search within titles and keywords employed iteratively the following search of terms: (port OR seaport OR terminal) AND (Sustainab*) AND (action OR measures OR policy OR sustainable development goals OR SDGs), until saturation was achieved. Search results yielded hundreds of studies. Studies were filtered in two stages based on inclusion and exclusion criteria, and those that reported repetitive result were excluded. Only abridged studies that are relevant in answering the study questions, and entailed scientific rigor were included, while there was no restriction on dates. To ensure the quality of the inclusion and exclusion criteria, 20 excluded studies were randomly selected to examine if their inclusion again would change the result, but no changes in themes were noted. Accordingly, 112 studies were included.

After included studies were collected, and then pre-explored, the literature was synthesised under various categories (typologies) that authors developed for port sustainability actions. While some typologies were already established in the field, the guidelines (Jaakkola 2020) for building typologies in a review paper were followed. The aim of building typologies is to explain differences between variants of a concept, categorise, organise fragmented research into common distinct types, and identify critical dimensions of a concept to reconcile conflicting findings from previous research (Jaakkola 2020). Thus, sixteen homogeneous and interconnected sustainability categories, including various measures, were identified, encompassing the Triple Bottom Lines (TBLs) of sustainability, i.e., the social, environmental, and economic dimensions. Additionally, the implementation schemes were divided into four groups. Also, the United Nation Sustainable Development Goals (UN SDGs) were presented to permit demonstration of the linkage between these UN SDGs and the port actions and measures in view of three dimensions of sustainability (TBLs). Whilst the literature is synthesised based on typology building, this critical review results in a conceptual framework, which generates new perspectives on the topic (Torraco 2005; Snyder 2019).

### Publications included in the review

This subsection overviews and brings in a summary of the features and characteristics of included studies. This adds more transparency to the study, enables readers to judge the coherence and plausibility of inferences, and enables future researchers to compare, contrast, build on, and update this database.

**Fig. 2:**
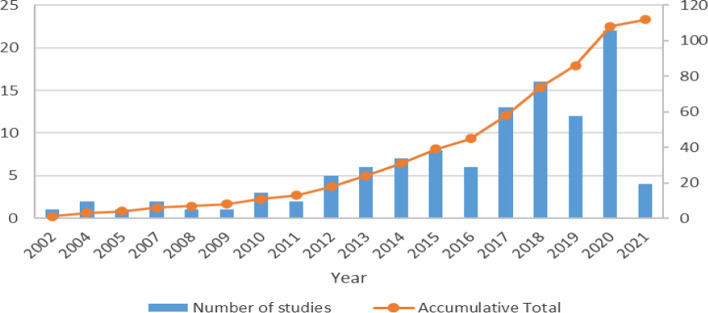
Trend of yearly publications

An amalgamation of 112 studies was included in this review. The trends of studies publication by year can be seen in Fig. 2. The port sustainability studies have increased significantly over the years. A considerable increase is noticeable from 2010 onward. This review includes different types of studies: 73 peer-reviewed articles (66%), 30 reports (26%), 5 book chapters (4%), and 4 conference proceedings (4%). Only 4 proceedings were included because many end up published in Journals and others are weak in context, while reports are mainly from the EU Commission including the European Seaport Organisation (ESPO), the International Association of Ports and Harbours (IAPH), and the World Port Sustainability Program (WPSP). The international Maritime Organisation (IMO) published several environmental-focus studies connoting the importance of a clean ship-port interface, while the United Nations Conference on Trade and Development (UNCTAD) addressed more of the economic aspects. However, the peer-reviewed articles are published in 32 different journals, and more than 65% of the studies are published in the following journals:

- Transportation Research Part D: Transport and Environment (8)
- Research in Transportation Business and Management (7)
- Maritime Policy and Management (7)
- Ocean and Coastal Management (6)
- Marine Pollution Bulletin (5)
- Sustainability (4)
- Journal of Cleaner Production (4)
- Energy Policy (2)
- Marine Policy (2)
- Maritime Technology and Research (2)

**Fig. 3:**
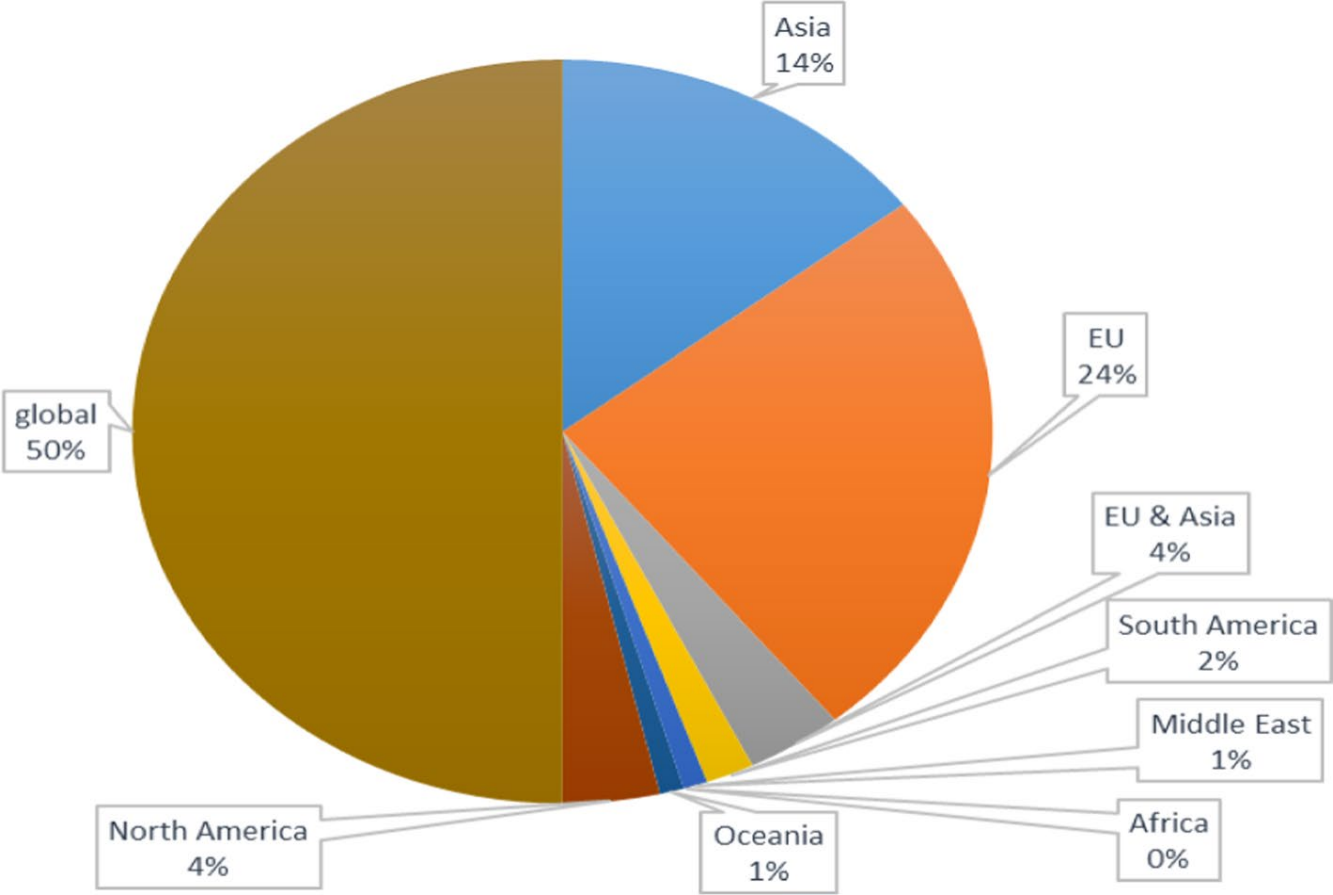
Precentage of studies' regional contribution

It is very noticeable that Transportation Research Part D, Research in Transportation Business and Management, and Maritime Policy and Management journals are publishing much of the research relevant to port sustainability. This aligns with Bjerkan and Seter (2019), Davarzani et al. (2016), and Stein and Acciaro (2020), who highlighted the same results. The rest of the studies are published by 25 journals, with one study per journal. These Journals cover a wide variety of topics, such as environment, transport, management, policy, engineering, sustainability, and energy. Methodologies utilised in the included journals’ studies vary, i.e., theoretical and conceptual including reviews (40%), simulation and modelling (24%), and case studies (20%), while only 16% are empirical (e.g., survey questionnaire and interviews). In terms of regional coverage of studies, as can be seen in Fig. 3, though 49% of studies are global in nature, the greatest density of studies is about EU countries (24%), and Asia (14%). This could be attributed to the strict regulations in EU and the large throughput of goods in Asian ports, which stimulate research in these regions. Africa, South America, the Middle East, and Oceania, on the other hand, are rarely introduced in studies.

## Literature review: building a port sustainability framework

### Sustainability and UN SDGs

Sustainability is not a new issue; it was addressed some time ago. In essence, the United Nations set up the UN Environment Programme (UNEP) in 1972, then the World Commission on Environment and Development produced a report called ‘Our Common Future: A Global Agenda for Change’, best known as The Brundtland Commission Report (1987) (WCED 1987). The report defined sustainable development as “the development that meets the needs of the present without compromising the ability of future generations to meet their own needs”. Generally, sustainable development includes three pillars [Triple Bottom Line (TBL)], i.e. economic, social, and environmental sustainability (Basiago 1999). Most academics and practitioners refer to sustainability as a concept that connotes the improvement and sustainment of environmental (ecological), economic, and social systems for humans (Stoddart et al. 2011; Mensah and Enu-Kwesi 2018). In other words, sustainability transforms and expands environmentally based concepts to involve social and economic aspects (trade-off) (Koberg and Longoni 2019). All in all, sustainability management systems certifications exists, e.g. environmental (ISO14001), and social (ISO26000) certifications (Koberg and Longoni 2019).

The United Nations Sustainable Development Goals (UN SDGs)—2030 Agenda—include various targets and indicators. The agenda was piloted by the principles of the United Nations Charter with due consideration of international law. It integrated previous agendas such as the Millennium Declaration and the 2005 World Summit Outcome Document. Seventeen SDGs were introduced to incorporate efficient solutions to inherent issues in the previous agenda (Table 1) (United Nations 2015). In this study, association between port sustainability actions and measures and these SDGs is introduced.

**Table 1:** United Nations Sustainable Development Goals (UN SDGs) of the 2030 agenda. *Source*: United Nations (2015)

SDG	Objective
Goal 1	End poverty in all its forms everywhere
Goal 2	End hunger, achieve food security and improved nutrition and promote sustainable agriculture
Goal 3	Ensure healthy lives and promote well-being for all at all ages
Goal 4	Ensure inclusive and equitable quality education and promote lifelong learning opportunities for all
Goal 5	Achieve gender equality and empower all women and girls
Goal 6	Ensure availability and sustainable management of water and sanitation for all
Goal 7	Ensure access to affordable, reliable, sustainable and modern energy for all
Goal 8	Promote sustained, inclusive and sustainable economic growth, full and productive employment and decent work for all
Goal 9	Build resilient infrastructure, promote inclusive and sustainable industrialization and foster innovation
Goal 10	Reduce inequality within and among countries
Goal 11	Make cities and human settlements inclusive, safe, resilient and sustainable
Goal 12	Ensure sustainable consumption and production patterns
Goal 13	Take urgent action to combat climate change and its impacts*
Goal 14	Conserve and sustainably use the oceans, seas and marine resources for sustainable development
Goal 15	Protect, restore and promote sustainable use of terrestrial ecosystems, sustainably manage forests, combat desertification, and halt and reverse land degradation and halt biodiversity loss
Goal 16	Promote peaceful and inclusive societies for sustainable development, provide access to justice for all and build effective, accountable, and inclusive institutions at all levels
Goal 17	Strengthen the means of implementation and revitalize the global partnership for sustainable development

### Port sustainability settings

Port operations include logistics functions (e.g., transport, terminal handling, warehousing and storage activities) in addition to industrial functions (e.g., goods and energy production, assembly, and disassembly and recycling activities) (Notteboom et al. 2020). To fulfil that, ports use various cargo handling equipment (CHE), for example, the ship-to-shore (STS), rubber-tired gantry (RTG), and rail-mounted gantry (RMG) cranes, yard trucks and tractors, top picks, side picks, handlers, forklifts, straddle carriers, chassis, reach stackers, and sweepers for container handling, pumps for liquid bulk ships, and loaders, dozers, cranes, and forklifts for bulk handling (Bailey and Solomon 2004; IAPH 2008; PIANC 2014; IMO 2018a). Furthermore, ports use vehicles and shuttles for local transfer, and storing cargo in warehouses and storage, and provide nautical services for calling ships through tug, pilot, and towing boats. Notably, most of these operations depend on fossil fuel, and consume energy, and thus operations generate environmental and social (employees, society, community, customers) externalities. In the same category, interaction of transport chains with ports generates various ecological, environmental and social impacts, such as the activities of ships (e.g., inland waterways, domestic, and oceangoing), inland trucks, railways and locomotives. Liquid bulk ships may bring the risk of oil spills, while cruise ships generate large amount of sewage and trash. Such issues would cause environmental deterioration if not monitored, controlled and treated sustainably.

To minimise port externalities, port sustainable management is the appropriate step. Port sustainability is defined as the business strategies and activities that meet the current and future needs of the port and stakeholders while protecting and sustaining human and natural activities (Denktas-Sakar and Karatas-Cetin 2012; Oh et al. 2018). Nevertheless, ports need to recognise that their actions today affect and influence the lives of future generations and the environment we live and work in. Thus, ports operate sustainably only when decisions are made based on long-term economic health and reflecting a profound and comprehensive devotion to environmental stewardship, while integrating community aspirations into business (I2S2 2013). Therefore, port sustainability covers much more than strictly environmental (planet) issues, i.e. it includes the triple bottom lines (TBLs) concept which extends the frame of sustainability to include social (people) and economic (profit) aspects (PIANC 2014; Lim et al. 2019).

Often, differentiation among these TBLs might not be clear. Generally, the economic sustainability dimension can be easily understood, i.e., generating positive financial gains. As regards the environmental sustainability dimension, it includes reduction of environmental externalities, such as waste and pollution reduction, improving energy efficiency and emission reduction, in addition to decreasing both the consumption of hazardous/harmful/toxic materials and the frequency of environmental accidents (Gimenez et al. 2012). Just as importantly, environmental sustainability also reduces social externalities, e.g., health problems, noise, safety risks—the bad side effects for communities and societies. The social dimension, on the other hand, focuses on the good sides for both internal employees and external communities, thereby providing equitable opportunities, encouraging diversity, improving community connectedness, and engaging in corporate social responsibility (CSR), among others (Elkington 1994).

Current research introduced the TBLs of port sustainability. Lim et al. (2019) demonstrated the interaction of TBLs indicating that they all interact together or in pairs. Port environmental sustainability minimizes the harmful impact that stems from port operation, ships, and land transport. Social sustainability improves the quality of employees’ lives and of neighbouring communities. Economic sustainability boosts port economic performance as a consequence of sustainability implementation while maintaining environmental and social sustainability (Lim et al. 2019). That being said, sustainability dimensions are interconnected, and thus cannot be pursued separately. Social issues may be influenced by environmental issues, and environmental aspects might be improved by ports’ economic support (Shiau and Chuang 2013). A case in point is the modal split measure^3^ which targets the reduction of trucks’ emission and congestion; it reduces CO_2_ emissions and air pollutants (environment), improves efficiency by reducing time and wasted efforts (economy), and eventually contributes to health and safety by decreasing accidents and fatalities and improving port employees and community health (social). Port economic sustainability (financial capability) is considered a driver for better environmental and social sustainability. Contrary to smaller ports, large ports which have economic sustainability are able to implement environmental and social measures due to funding availability (Kuznetsov et al. 2015). For example, the ports of Antwerp, Hamburg, Los Angeles, Rotterdam and Vancouver have accomplished substantial local air quality advances, even though general cargo throughput has increased (Poulsen et al. 2018).

It should be noted that maritime transport is a nexus of the global supply chains (Asgari et al. 2015), and it has been shown that ports have a role to play in green supply and global value chains (Poulsen et al. 2018; Notteboom et al. 2020). It is crucial that ports implement sustainability measures in collaboration with the key members of the supply chain (Lu et al. 2016b), i.e. the shipping lines, ocean carriers, freight forwarders, rail operators, and trucking companies*.* Collaboration in this case advances operational efficiencies and improves port sustainability (Seuring and Müller 2008; Kang and Kim 2017; Poulsen et al. 2018; Alamoush et al. 2020). On another note, WPCI members have claimed that ports can influence the sustainability of supply chains as they occupy a distinctive location and act as key hubs in global supply chains (WPCI 2010). Hence, the outreach of port sustainability should also be of consideration; that is, implementing actions and measures to yield sustainable transport and supply chains. While ports take actions internally, i.e. relevant to inland port operations through the internal sustainable management, external sustainable management (external actions) including supply and transport chains, is as important as internal sustainable actions (Lu et al. 2010; Denktas-Sakar and Karatas-Cetin 2012; Yang and Chang 2013; Lu et al. 2016a; Lu et al. 2016b; Roh et al. 2016). Through external sustainability management, ports expand the sustainability concept from the port itself to the supply chain activities beyond its boundary.

**Fig. 4:**
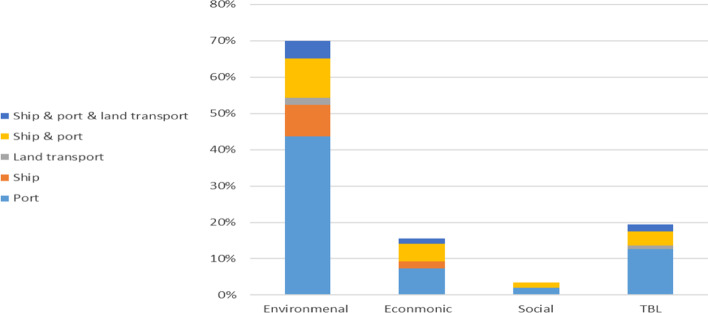
Percentage of studies coverage by dimensions of sustainability and internal and external scope

Drawing from the literature review, extant research has actively presented various aspects of port sustainability, but still various gaps exist. Firstly, as can be seen in Fig. 4, previous research greatly focused on environmental aspects of sustainability (65%), and sometimes modestly integrated the economic (14%) and social (3%) aspects. Similarly, only 17% of studies covered the TBLs of sustainability including technical reports. Although building green ports is now a common practice to enhance environmental sustainability, the social aspect of sustainability is always addressed less in the literature (Shiau and Chuang 2013). The important social dimension of port sustainability considers employee issues, stakeholder relationships (e.g. carriers and stevedoring companies), ethical issues, and corporate social responsibility (Oh et al. 2018). Only recent research has demonstrated port sustainability by addressing the environmental dimension including the social and economic aspects or alternatively the TBLs (Table 2).

**Table 2:** Port sustainability studies, TBLs dimensions, and scope

Study	Objective	TBLs	Scope
Peris-Mora et al. (2005)	Studied the potential 63 environmental impacts in ports and established 17 environmental indicators	Envi	Int
Darbra et al. (2009)	Studied the EU ports environmental issues and presented the self-diagnosis method (SDM)	Envi/soc	Int
Lam and Van De Voorde (2012)	Built a framework for green port strategy	Env/Eco	Int/Ext
Lirn et al. (2013)	Examined green port performance criteria (externalities) and presented 17 green performance indicators	Envi & soc	Int
Yap and Lam (2013)	Studied container ports’ spatial planning and development and presented the impact on port sustainability	Env/Soc	Int/Ext
Shiau and Chuang (2013)	Studied the sustainability indicators (case study of Keelung port-Taiwan) and identified 34 sustainability indictors	TBL	Int
Lam and Notteboom (2014)	Presented port authorities’ green tools in leading Asian and European ports (green ports)	Envi	Int/Ext
Acciaro et al. (2014)	Assessed the success of ports’ innovations in terms of environmental sustainability	Envi	Int
Chiu et al. (2014)	Studied green port operation and revealed five green priorities	Envi & soc	Int
Puig et al. (2015)	Studied Environmental issues in European ports and provided a Tool for the identification and assessment of Environmental Aspects in Ports (TEAP)	Envi	Int
Asgari et al. (2015)	Ranked the UK ports’ sustainability based on nine criteria and five sub-criteria	Envi & Eco	Int
Davarzani et al. (2016)	Reviewed green ports and maritime logistics	Envi	Int
Sislian et al. (2016)	Literature reviewed port sustainability	TBL	Int
Roh et al. (2016)	Studied the internal and external management aspects of sustainable ports based on six management criteria	TBL	Int/Ext
Lu et al. (2016b)	Assessed the ports sustainability criteria and reports	TBL	Int/Ext
Santos et al. (2016)	Investigated sustainability communication practices in the European seaport sector	Soc	Ext
Puig et al. (2017)	Studied Environmental issues in European ports and Provided a Tool for Identification and Implementation of Environmental Indicators (TEIP)	Envi	Int
Laxe et al. (2017)	Development of port sustainability “global synthetic indicators”, based on 9 indicators	TBL	Int
Oh et al. (2018)	Presented the criteria for assessing sustainability of ports in south Korea, and Identified 27 sustainability assessment items	TBL	Int
Lim et al. (2019)	Reviewed and synthesised port operational and management indicators for sustainability based on 30 indicators	TBL	Int/Ext
Bjerkan and Seter (2019)	Reviewed and structured port sustainability in port management and policies, power and fuels, sea activities, and land activities	Env	Int/Ext
Notteboom et al. (2020)	Presented ports’ role in the pursuit of green supply chain management through five actions	Env	Int/Ext
Hossain et al. (2020)	Investigated global ports’ implementation of sustainability initiatives	TBL	Int
Castellano et al. (2020)	Evaluated the relation between port environmental quality and economic efficiency (Italian Ports)	Env/Eco	Int
This study	Develops a port holistic sustainability framework that integrates TBLs in internal and external actions and measures while drawing an association with UN SDGs	TBL and UN SDGs	Int/Ext

Int, internal, Ext, external

Secondly, the sustainability outreach (scope) (Fig. 4) is included in most cases, i.e., either internally for the port side, or/and externally including ships, except the land transport, while the main focus is still on the internal actions (port side). As can be seen in Fig. 4, 54% of studies addressed the port side only (internally), followed by port side and ships (26%), ships (12%), and port side, ship, and land transport (7%), while land transport alone is rarely included in studies (1%). Another issue in the sustainability scope from a dimensional perspective is that TBLs do not address the external scope extensively. Put differently, only very few external actions and measures are presented. For example, but not limited to, no studies addressed the social aspects of seafarers, truck drivers, collaboration with supply chain members, partnerships with academic institutions, and public participations in environmental project planning, etc.

Thirdly, an important note which can be gleaned from reviewed studies is that some studies mixed the actions and measures (technical and operational) with institutional, management and policy tools (called implementation schemes in this study). Although not highly discussed, the implementation schemes are tools introduced as an independent form of governance to formulate policies that guarantee development and uptake of sustainability actions and measures (Laxe et al. 2017; Bjerkan and Seter 2019). Furthermore, there is no one study that included all the actions and measures internally and externally while at the same time integrating the TBLs dimensions. In other words, results are fragmented, and, if an action appears in one study, it doesn’t necessarily appear in another.

Fourthly, chief among observations is that no study attempted to link port sustainability actions with the TBLs dimensions and with the UN SDs (see Table 2). Though, a few studies briefly pointed out that port sustainability measures are foundations to the SDGs, e.g., (Alamoush et al. 2020; Notteboom et al. 2020; WPSP 2020). A holistic investigation of ports’ contribution to UN SDGs is scarce, thus, this is one of the main gaps this study aims to fill. Nonetheless, it is worth noting that other maritime and marine research has addressed SDGs. Notable examples are: investigation of marine spatial planning as a process to achieve SDGs (Pyć 2019), study of coastal and marine conservation strategies (in Bangladesh) in the context of achieving blue growth and SDGs (Islam and Shamsuddoha 2018), connecting SDG 14 (life below water) with the other SDGs from a marine spatial planning perspective (Ntona and Morgera 2018), mapping the linkages between oceans (SDG 14) and other SDGs (Le Blanc et al. 2017), and development of port sustainable supply chain management frameworks to achieve the SDGs (Alamoush et al. 2021a).

To facilitate locating the relevant literature (peer-reviewed), Table 2 summarises chronologically key studies that addressed the port sustainability including the scope (internally and externally), and the TBLs dimensions.

Academic research and the international frameworks that address port sustainability are equally important. The International Maritime Organisation (IMO) has established guidelines regarding measures to reduce ships’ (IMO 2015) and ports’ (IMO 2018b) emissions, and produced four IMO greenhouse gas studies (ships), the most recent is the fourth GHG study (IMO 2020c). The World Port Climate Initiative (WPCI) and the International Association of Ports and Harbours (IAPH) have established guides on port environmental measures, GHG emission reduction and carbon footprinting, onshore power supply, and the testing of innovative cargo handling equipment (CHE) (IAPH 2007, 2008; WPCI 2010). WPCI was expanded in line with World Ports Sustainability Program (WPSP), which is a joint initiative with the IAPH. The WPSP issued the World Ports Sustainability Report in 2020, which included ports’ contribution to the SDGs (WPSP 2020). The American Association of Port Authorities (AAPA) produced an environmental management book as a guide for North American Ports, and the European Seaport Organisation (ESPO) is an active regional organization for European ports. ESPO, based on the EcoPort initiative, listed the common port environmental management priorities, i.e. air quality, energy consumption, climate change, noise, relationship with local community, ship waste, garbage/port waste, port development, dredging operations and water quality (ESPO 2019). Finally, the World Association for Waterborne Transport Infrastructure (PIANC)^4^ (PIANC 2014) and International Institute for Sustainable Seaports (I2S2)^5^ (I2S2 2013) produced reports about ports’ environmental initiatives from a global perspective.

**Fig. 5:**
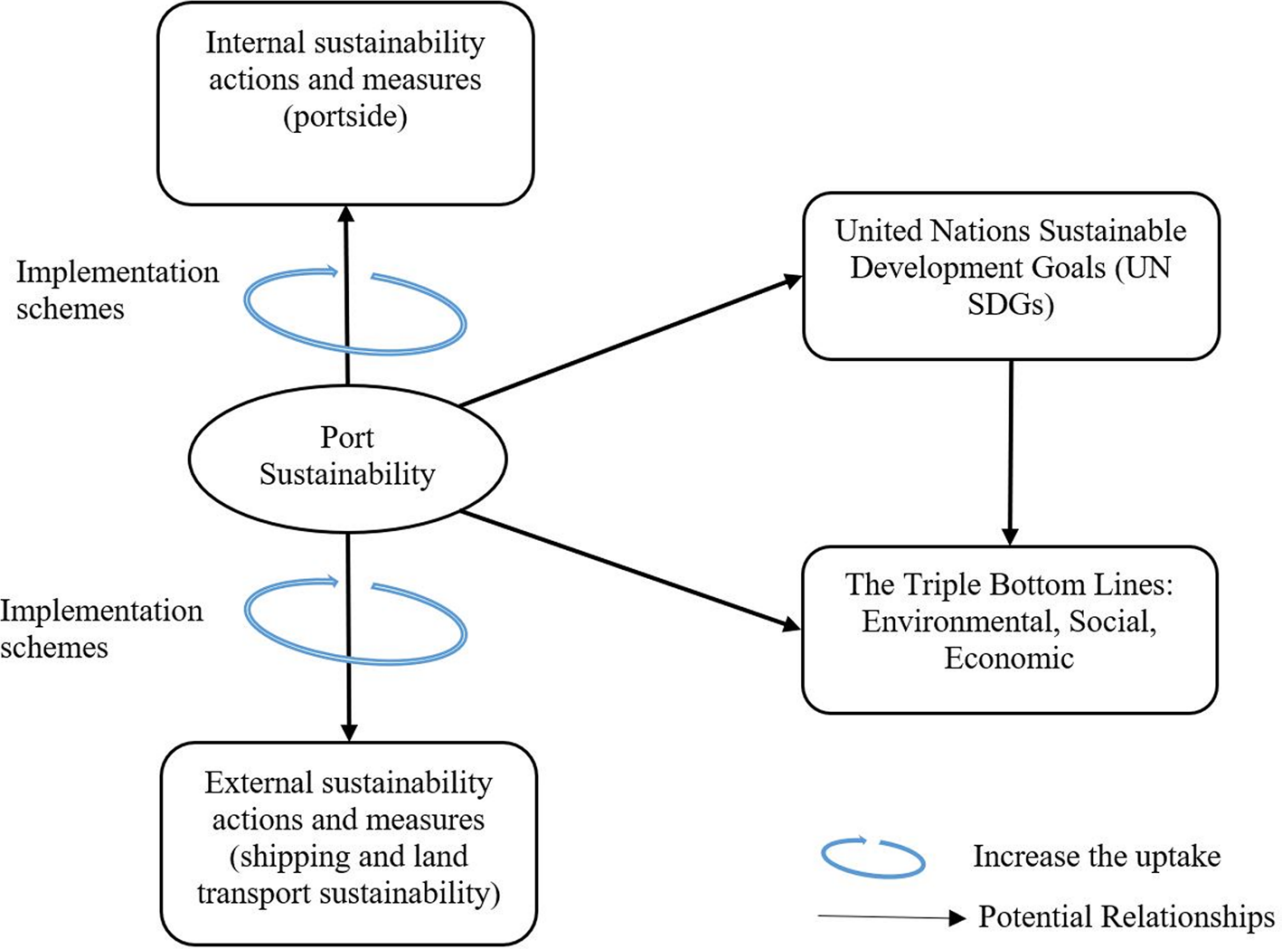
Study’s conceptual framework

Figure 5 below demonstrates the study’s conceptual framework, which summarises our findings thus far and illustrates the concept of port sustainability with presumed relationships, noting that results and discussions in this study are reported according to this framework. Conceptual frameworks are customarily generated within critical/integrative literature reviews (Yadav 2010; Jaakkola 2020). As can be seen in Fig. 5, port sustainability encompasses the triple bottom lines (TBLs), i.e., by taking actions and measures to mitigate and eliminate the port environmental externalities (protecting the integrity of the environment) and improve the social aspects (employees, labour and communities), while at the same time endeavouring to strengthen port economic benefits. Actions and measures span the internal port operations, and expand externally to include the main transport chains (mainly ships and trucks). Furthermore, implementation schemes work as catalysts that increase the uptake and prompt operationalisation of measures and actions. Like the linkage with TBLs, port sustainability is proposed to be linked to UN SDGs, which are also linked with the TBLs. Against this background, this study fills all the identified gaps in previous studies and adopts and captures broader and more actions and measures of port sustainability than any previous study by identification 16 actions along with 138 measures that achieves these actions, in addition to four groups of implementation schemes. The measures are either tabulated or explained within the text. It is worth noting, however, that not all the actions and measures are implemented in ports, and thus they are proposed to advance port sustainability. The same is true with regard to the implementation schemes, they are also proposed to advance implementations. Last but not least, the linkage between port sustainability and the UN SDGs is identified.

## Internal and external ports’ sustainability actions and measures and the association with SDGs

This section includes the three dimensions of port sustainability actions (i.e., the environmental, social and economic dimensions) in addition to the implementation schemes.

### Environmental dimension actions

Ports’ actions to minimise environmental externalities are overarching and highly covered in the literature. The environmental measures and actions are adopted in environmental management systems, which are accredited and reported in different schemes. Examples are the ISO14001 environmental management system, the European Union’s eco-management and audit scheme (EMAS), and ESPO self-diagnosis method (SDM), and port environmental review system (PERS)—the EcoPort initiative—which incorporates the generic requirements of the environmental management standards (e.g. ISO 14001). PERS is more common in EU ports than the ISO standards which is common in Asian ports (Darbra et al. 2009). Some ports have specifically addressed energy management and audit through the certification acquired through ISO50001, e.g. Ports of Antwerp, Valencia, Rotterdam, Genoa, Dover, and Livorno (ESPO 2018). The environmental actions aggregated into homogeneous categories follow.

### Air pollution management

The air quality issue is a primary port externality, which is worsened by the dusts from traffic, site clearing, rock excavation and construction activity. Likewise there are the ambient air emissions (pollutants) from port traffic and operations, i.e. oxides of sulphur (SOx), oxides of nitrogen (NOx), particulate matter (PM), carbon monoxide (CO), and volatile organic components (VOC) (Gupta et al. 2002; Ng and Song 2010; Lam and Notteboom 2014; Roh et al. 2016)*.* The air pollutants in ports generate environmental and social impacts (externalities) (Dinwoodie et al. 2012). The environmental impacts include the ocean acidifications, inter alia. Socially, they affect the health of employees and local communities causing adverse health problems such as respiratory diseases (asthma), cardiovascular disease, lung cancer, premature death, and birth defects (Bailey and Solomon 2004; Chang and Wang 2012; MTCC Pacific 2017; IMO 2018a). Corbett et al. (2007) estimated that about 60,000 annual cardiopulmonary and lung cancer deaths along the European, East Asian, and South Asian coastlines are due to particulate matters (PMs) emissions from commercial ships. Another port, shipping, and truck air emission, associated with climate change (global warming) and ocean acidification, is GHG such as carbon dioxide (CO_2_) emissions (Ölçer et al. 2018; Alamoush et al. 2020).

Therefore, to reduce air pollution—removing the environmental externalities that also reduce social externalities—ports can take actions (shown in Table 3) to: reduce ambient air emissions and limit and decrease dust and odour. In the same table, a non-exhaustive list of measures that operationalise each action is presented. The measures span port wide related operations in addition to shipping and land transport. It should be noted that some of the air emission reduction measures may have co-benefits with the reduction of GHG emissions, but specific measures to mitigate GHG emissions are addressed separately under the climate change mitigation and adaptation action.

**Table 3:** Air pollution management actions and measures. *Source*: Peris-Mora et al. (2005), Darbra et al. (2009), Ng and Song (2010), Dinwoodie et al. (2012), Chang and Wang (2012), I2S2 (2013), Lirn et al. (2013), Shiau and Chuang (2013), PIANC (2014), Acciaro et al. (2014), Roh et al. (2016), IMO (2018b) and Oh et al. (2018)

Areas for action	Measures
Air emission reduction^a^	Establish emission inventory and energy consumption
	Monitoring of CHE, ships’, and trucks’ emissions
	Replacement of polluting equipment or engine exchange (with cleaner ones)
	Electrification, hybridisation of CHE (e.g., electric RTGs for containers and shore-side pumps for bulk liquids)
	Use of emission reduction/control technology (pre-after treatment retrofit), such as the Diesel retrofit technologies (Diesel Oxidation Catalysts (DOC), Diesel Particulate Filters (DPF) or Selective Catalytic Reductor (SCR))
	Use of low-sulphur fuel and renewable alternative fuels (hydrogen, LNG, ammonia, renewable diesel and methane)
	Promote public and environment-friendly transport (employees’ sustainable mobility through shuttle bus, carpooling, cycling)
	Onshore power supply (OPS) for ships (e.g., for energy intensive cruise and containers ships), and tugboats and pilot boats when stationary and idling
	Providing power supply (charging stations) for electrified trucks
	Provision of alternative fuel bunkering for ships (e.g., LNG)
	Reduce truck congestion (e.g., using off-dock staging yards and chassis, building dry ports and inland depots, manging truck empty return, and utilising the Authorized Economic Operator System (AEO), automatic clearance and extended gate hours)
	Reduce trucks’ emissions through ban of old trucks, terminal appointment system (TAS), truck identity card, traffic mitigation fees, and off-peak traffic shift
	Enforce modal split (from road to rail, inland waterways and pipeline)
	Manage motorways of the seas (MoS)
Dust and odour reduction	Utilise dust and smoke recycle measures (e.g., for dry bulk ships)
	Build physical barriers to stop/reduce dispersion of air pollutant (e.g., tree belts, walls)
	Minimise Volatile Organic Components (VOC) emitted during loading and unloading operations (liquid bulk ships)

^a^Air emission reduction measures particularly reduce ambient air emissions; however, they generally reduce GHG emissions, thereby contributing to climate change mitigation actions—except the DOC, DPF, and SCR that may increase energy consumption

### Water pollution and waste management

Ports’ locations and their maritime accesses are typically situated near communities and natural habitats and species. As mentioned earlier, port operations and related supply chain activities create multifaceted impacts. Within the port, some operations and activities degrade the sea’s water quality (pollution), e.g., sewage, bilge wastes, sludge waste, oil discharges, dredging, and leakages of harmful materials (Gupta et al. 2002; Peris-Mora et al. 2005; Darbra et al. 2009). On the other hand, port waste contaminates soil and ground water and poses environmental, health and safety risks, and dredging causes water quality issues pertaining to turbidity and endangered species (PIANC 2014).

Further water pollution can be caused by ships’ oil spills, ballast water, cargo residue and garbage discarding (Peris-Mora et al. 2005; Ng and Song 2010; Dinwoodie et al. 2012; Lirn et al. 2013), which damage beaches and soils, and endanger marine habitats and wildlife. Shipping ballast waters introduce alien species into national waters which can negatively impact marine ecosystem health, devastate natural species and consequently generate an ecological imbalance, in addition to generating negative impacts on human health and marine resources economics (loss of profit) (Lirn et al. 2013). Even ships’ sewage, if disposed into the sea within the port areas, can provoke skin diseases as well as having impacts on the underwater environment and habitats.

Therefore, ports can prevent and minimise disposal of effluents, and water pollution, and maintain standard water quality. Measures which can be taken are various, among others, to control, prevent and monitor spill of cargo and oil during loading and unloading and disconnection of pipelines (liquid bulk ships), and from engine oil and lubricants (Laxe et al. 2017). Sewage tanks can be sealed and monitored. Stormwater runoff from cargo handling operations can run directly into adjacent waters, therefore, swales, storm filters, cyclonic devices and planters can be utilised to improve stormwater runoff quality (I2S2 2013; Roh et al. 2016). Port low impact design (LID) was included in the stormwater management programs, e.g., in the U.S ports (I2S2 2013).

Ports’ regular waste needs to be separated and classified along with litter control mechanisms (Ng and Song 2010). On the other hand, for ships, ports provide ballast treatment facilities, and reception facilities (sewage treatment), including trash. This is important for cruise ships as they generate large amount of sewage and trash. Ports introduce floating or mobile reception facilities with the ability to collect, classify and separate various types of ship waste (PIANC 2014). In addition, environmentally friendly services (e.g., ships’ hull and propeller cleaning) can be delivered, while, on the other hand, care should be taken to observe the standard of ship’s sanitation equipment (Ng and Song 2010; Dinwoodie et al. 2012). Oil and chemical spills, from liquid bulk ships, are common within and around ports. In this manner, oil spill contingency plans cover measures that should be taken to prevent, control, and respond to any spill. Spillages can be secured by deploying booms and skimmers (I2S2 2013; PIANC 2014).

### Noise pollution management

Sound pollution (noise) in ports, through cargo handling, construction, shipping, land transport and temporary dredging activities, reduces the quality of life and creates health hazards, in addition to ecological impacts, e.g., the adverse effects on marine mammals and fish (PIANC 2014; Enguix et al. 2019). The traffic generated around the port by movements of heavy duty vehicles and railways generates social and health impacts (e.g., noise, vibration, road congestion, and accidents) in surrounding communities, who usually complain about such issues (I2S2 2013; Lirn et al. 2013; PIANC 2014). ESPO rates noise in ports as one of the top environmental priorities (ESPO 2019). Nonetheless, it is necessary that ports take actions to monitor, limit, and mitigate noise above and under water. Measures that can be taken include: building noise maps; zoning of noisy activities; use of standards for limitation of noise and vibration from CHE and construction (e.g., isolation of forklifts, trucks, vehicles and tugs); insulation of windows, doors, and fences; building noise barriers around the port (e.g., concrete, trees, and earthen walls), and sound absorption materials on buildings and walls; use of silent asphalt and tyres; and planning of activities on the basis of meteorological conditions (wind direction) (I2S2 2013; PIANC 2014). Additionally, to protect against underwater noise, fish bubble curtains can be used to mitigate the noise of dredging (I2S2 2013). On the other hand, particularly for ships, ships’ noise can be monitored and characterised, using sonars, echo-sounders, robotics, and hydrophones (Enguix et al. 2019). Thus, ports can dedicate protected areas, buffer zones, and corridors to keep ships away from rich marine environments. Likewise, ports can implement slow steaming of ships and tugs (cavitation inception speed), and utilise air bubble curtain technology to absorb shipping noise (Domenico 1982; I2S2 2013; PIANC 2014; Enguix et al. 2019).

### Visual pollution (light and aesthetics) management

The unattractive appearance of port buildings, uncovered cargo stockpiles and high CHE disrupt landscape (visual impacts) and quality of life (PIANC 2014). Ports need to minimise this by appraising the visual impact of existent landscapes. For example, new facilities can take advantage of existing topography and maintain low profile infrastructure and equipment (PIANC 2014). Other measures can be applied, such as changing buildings’ colour schemes in addition to camouflage or disguise, and planting trees in landscaping buffer zones (aesthetic areas) (I2S2 2013). On the other hand, light pollution harms workers and neighbouring residents, but biological spectrum lighting can be used to mitigate negative impacts (Lirn et al. 2013; Chiu et al. 2014; Oh et al. 2018).

### Freshwater management

Water consumption in ports is high, specifically within operations, cleaning and washing bulk ships and yards, and the supply for highly consuming cruise ships. Measures to conserve water and protect freshwater resources can be established. For example, ports may set goals to reduce waste of drinking water, monitor water usage and leakage, treat and use waste water (on-site), recycle cleaning water for irrigation and cleaning, and harvest rain water (Lirn et al. 2013; Yang and Chang 2013; Laxe et al. 2017).

### Marine biology conservation

Marine biology is highly influenced by port operation, expansion and construction activities. Therefore, high attention should be paid to decreasing, monitoring and controlling the impacts on marine biology (flora and fauna). As can be seen in Table 4, ports can employ measures to limit sediments impact, avoid the destruction caused by dredging, protect habitat quality in water and above water areas, and control floods (Shiau and Chuang 2013; PIANC 2014; Roh et al. 2016). Dredging programs—for channel and berth deepening to accommodate larger ships—require innovative mitigation and stewardship of natural resources (e.g., sediment management plans) (I2S2 2013).

**Table 4:** Marine biology conservation actions and measures. *Sources*: I2S2 (2013), Lirn et al. (2013), Shiau and Chuang (2013), Yang and Chang (2013), Chiu et al. (2014), PIANC (2014), Roh et al. (2016), Laxe et al. (2017), Oh et al. (2018) and Lim et al. (2019)

Areas for action	Measures
Limit and treat sediment	Reuse of dredging sediments
	Control port entrance sediment and coastal erosion
	Deposit (dispose) sediments in a separated area
Avoid dredging destruction	Monitor dredging operations (pre and after dredging sampling)
	Source, lease and permit environmentally friendly dredgers
	Remediation of contaminated sites and mitigation of turbidity
Protect habitat quality (underwater and above water areas)	Ecological monitoring and mitigation in port areas for habitat quality, preservation, and wetland restoration
	Expansion of tidal areas for habitat restoration
	Creation of local sanctuaries for birds and fish in and around port areas
	Soil pollution monitoring
	Buying, creating, selling, and banking ecological service credits (i.e., wetland, grassland, and forest) to offset development impacts on wetlands
	Establishment of buffer zones for endangered coral relocation
	Fish bubble curtains along harbour entrances to keep fish out of the dredging area
	Monitor and control of ship’s fouling (antifouling), and discharge of effluents
Flood control	Prevention of floods by proper training and using innovative technologies

### Hazardous cargo management

In addition to following the International Maritime Dangerous Goods (IMDG) Code, ports implement measures for hazardous cargo ships and hazardous cargo handling, for example, separation of hazardous goods and construction materials, and employment of licensed contractors to handle hazardous waste (PIANC 2014; Roh et al. 2016; Laxe et al. 2017). Hazardous cargo negligence, including explosives and chemicals in bulks, has huge environmental impacts on societies and life below water. The huge explosion that occurred in Beirut port largely influenced the whole surrounding environment, which was due to issues in storage and separation of dangerous cargo. The explosion, in addition, negatively influenced employees, seafarers, ships and the port, socially and economically. Even recently, relevant to COVID-19, sterilizing and fumigation of cargoes coming from epidemic areas is an adopted measure to minimise the spread of contagions (Notteboom and Pallis 2020c).

### Climate change mitigation and adaptation

While ports are central nodes in global transport chains, they are exposed to climate change, particularly in view of their locations on coasts and shores, and their access points. Given their valuable contribution to economies, and the associated valuable infra/super structure; ports have a crucial role to play in climate change (UNCTAD 2017). Ports’ roles in climate change can be either by preparing to adapt to its future impact or by reducing its precursor (GHGs), i.e., adaptation and mitigation, respectively.

While climate change impacts are devastating, e.g., sea level rise^6^ and storm surges (cyclone, tornado), intense rainfall, and higher wind speeds, ports in return need to prepare by taking adaptation actions and measures (see Table 5) to remain operational. Otherwise, such impacts damage port infrastructure, and degrade port operation, thus leading to more downtime for cargo handling and clearance, and delays for ships and land transport (Wilmsmeier 2020). From a mitigation perspective, ports utilise measures to reduce GHG emissions (decarbonisation), including energy efficiency, in port landside operation, and facilitate the reduction of ships and land transport GHG emissions (see the measures in Table 5).

**Table 5:** Climate change mitigation and adaptation actions and measures. *Sources*: Villalba and Gemechu (2011), I2S2 (2013), Ng et al. (2013), PIANC (2014), UNCTAD (2017), Iris and Lam (2019), Alamoush et al. (2020) and Wilmsmeier (2020)

Areas for action	Measures
Adaptation actions	Building walls and beach restoration
	Protecting against coastal erosion
	Use of climate change monitoring applications
	Establishment of natural defences, e.g., planting mangroves, and creating oyster reefs that grow with sea level rise and protect shorelines and ports from high waves and erosion
	Consideration of climate sensitive designs
Mitigation actions (GHG reduction and energy efficiency)	Establishment of energy consumption inventory and carbon footprinting, including shipping and land transport
	Use of renewable energy technologies (wind, solar, ocean, geothermal)
	Energy consumption reduction through insulation, coating, and painting of buildings, storage, warehouses, and using reefer sheds
	Use of the after pre and after treatment technologies in CHE (e.g., Methane catalyst reductor)
	Design of energy efficient infrastructure through adopting the LEED standard for green building energy efficiency designs (passive house concept), and microclimate models
	Use of LED lights and automatic sensors
	Use of energy efficiency technologies (e.g., smart grids, microgrids, smart load management, regenerative energy reclamation, virtual power plants, energy storage systems, energy saving tyres)
	Eco driving, idle control and reduction, slow steaming, speed reduction
	Control of heat, ventilation, and air conditioning (HVAC)
	Operational efficiency planning (e.g., cranes and yard planning)
	Use of biomasses to generate power and heat
	Introducing carbon sequestration, capture and storage projects

Ports emit 3% of global GHG emissions (Misra et al. 2017), and shipping emits 2.89% (1076 million tonnes in 2018) (IMO 2020c). Five percent of shipping GHG emissions are in ports areas (ITF/OECD 2018), which roughly account for 50% of ports-related emissions (Winnes et al. 2015). Taking the Port of Rotterdam container terminals as an example, its share of CO_2_ emission represents 2% of total CO_2_ emissions of Netherlands freight transport (Geerlings and van Duin 2011). Obviously, unless shipping and ports take measures to reduce emissions, shipping GHG emissions are expected to increase by 90–130% by 2050 compared to 2008 levels (IMO 2020c). The IMO^7^ has reacted and initiated the Initial GHG Strategy to reduce shipping GHG emissions (IMO 2018c), and even called for ports to facilitate shipping emission reductions (IMO 2019).

### Circular economy

Port operational and industrial activities and infrastructural development use and generate large volumes of material at sea and on land, which, if not controlled, will create environmental externalities. Therefore, ports can close the material loop by introducing recycling, so as to avoid significant waste flows (PIANC 2014). Circular economy approaches can be significantly adopted in ports, for reducing, recycling, and reusing waste, and thus reach out to change the supply chain to circular rather than linear states (de Langen and Sornn-Friese 2019). The reduce-reuse-recycle measures, in-house or outsourced through integration with the city, are across-the-board. Notwithstanding that, the circular economy may offer profitable business cases. Thus, a port can recycle office waste, paper, dunnage, glass, metals and plastics, engines oil and lubricants. In addition, ports may reuse construction waste materials, recycle materials to be used for buildings, facilities and construction, and reuse heat and steam from port industries (Acciaro et al. 2014; de Langen and Sornn-Friese 2019; Alamoush et al. 2020).

### Social dimension actions

Social actions in ports are of paramount importance. While being socially sustainable, ports take action—internally and externally—to improve issues regarding employees, community, supply chain members and stakeholders. Social actions have been aggregated (Table 6) to encompass employees’ rights, safety and security, community and seafarers. As can be seen in Table 6, various measures can be utilised to realize relevant actions, thereby improving the welfare of employees, decreasing accidents and socially engaging and supporting the community. For example, vocational training in port skills for low-income young people (community) aims at social inclusion and, in so doing, enhances logistic careers for youth in the region. Furthermore, ports as a hub contribute to the employment of communities’ personnel. Just in the port of Antwerp in Belgium, 142,348 people were employed in 2015, of which 60,656 were directly employed (Esser et al. 2020). Open and transparent sustainability reporting is a positive measure ports take towards showing the community their robust stance in corporate responsibility (Santos et al. 2016; Hossain et al. 2020). Port sustainability reports exist, and typically include environmental and social actions and measures, e.g., Port of Antwerp sustainability report (Port of Antwerp 2017).

**Table 6:** Social dimension actions and measures. *Sources*: Shiau and Chuang (2013), Lu et al. (2016b), Sislian et al. (2016), Santos et al. (2016), Roh et al. (2016), Laxe et al. (2017), Oh et al. (2018), Lim et al. (2019) and IMO (2020b), IMO (2020d)

Areas for action	Measures
Employees rights	Improvement of employee’s welfare and health
	Non-discriminative employment
	Ensuring gender equality and diversity in employment
	Provision of continuous training and education
	Maintaining employees’ job security
Safety and security	Monitoring, control and minimisation of accidents and near miss incidents
	Improvement of work security and safety
	Implementation of ISPS code
	Preparation of disasters and incidents contingency plans
	Preparation of hazardous and dangerous materials storage plans, e.g. safe cargo handling according to IMDG Code^a^
	Improvement in safety of infrastructure and roads
	Ensuring safe and secure navigation for ships
	Collaboration with supply chain members to minimise risks, and improve safety
Community	Support of local employment (job opportunities)
	Encouragement of public participation in port environmental projects planning
	Recognising the requirements of the neighbouring community (e.g., via public opinion survey)
	Managing visual impact and improving city scenery^a^
	Mitigation of value decrease in community real estate because of repellent operations (e.g. cargo pipelines, stockpiles, noise)^a^
	Expanding corporate social responsibility (CSR) to include communities (e.g. provision of scholarships, internships, and vocational training for locals, offering local tours, supporting economically local projects and tourism industry development)
	Partnership with academics/research institutions, e.g., for project evaluation
	Reporting of port sustainability through (GRI guidelines) and/or in port website
Seafarers	Facilitating seafarers’ welfare by permitting port and city calls
	Facilitating crew changes and repatriation
	Ensuring seafarers rights are well taken care of on board calling ships

^a^Discussed earlier within the environmental section, but still, it is essential for safety and security, and community

Notably, seafarers’ social issues should not be neglected in port actions towards social satiability, as two million seafarers operate the global shipping fleet (IMO 2020b). This issue of seafarers was brought to the fore during the COVID-19 pandemic: many seafarers suffered due to restrictions on travels, ports banning embarkation and disembarkation, including city calls, quarantine measures, and limits on the issuing of visas and passports, leading to a crew change crisis (i.e., 300,000 seafarers were trapped working aboard ships) (IMO 2020d).

### Economic dimension actions

Economic sustainability enhances port economic performance (Oh et al. 2018). While port economic actions maintain port profitability, and facilitate trade, it goes without saying that such actions uphold environmental and social sustainability (Lim et al. 2019). For example, improving efficiency within the port logistics chain decreases CO_2_ emissions (Alamoush et al. 2020).

Economic actions and measures (see Table 7) are diverse (internally and externally). Although they are interconnected, an attempt is made to aggregate them into: economic growth, trade and logistics facilitation, and digitalisation actions. Measures such as investment in port infrastructure, and attracting foreign investment improve port profitability and maintain competitive advantage (Shiau and Chuang 2013; PIANC 2014; Asgari et al. 2015). In addition, linked to economic growth, the trade facilitation measures improve the economic advantages of supply chains and stakeholders, and thus render their operation cost efficient (Lim et al. 2019) (Yap and Lam 2013). Given the need to continue trade and facilitate ships’ berthing and handling while keeping social distancing measures or teleworking during the COVID-19 pandemic; digitalisation measures (technologies) are considered top priority for ports and the whole of maritime transport. Digitalisation can help resuming cruise business, for example in checking health certificates in passengers and cruise ships, considering that handling thousands of passengers’ certificates manually complicates getting back to normal operations. However, the growing cyber risks due to dependence on Information Communication Technologies (ICT) has recently increased in ships and ports (UNCTAD 2020b). It should not be ignored that the cyber risk would disrupt operations and may even shut down the whole port. Therefore, cyber security measures are essential to advance secure digitalisation.

**Table 7:** Economic dimension actions and measures. *Sources*: Yap and Lam (2013), PIANC (2014), Asgari et al. (2015), Lu et al. (2016b), Roh et al. (2016), Laxe et al. (2017), Bjerkan and Seter (2019), UNCTAD (2019b), Alamoush et al. (2020), Pu and Lam (2020) and Yap and Lam (2020)

Areas for action	Measures
Economic growth	Investing in port infrastructure
	Establishing port development funds
	Attracting foreign investment (public private partnership (PPP), concessions)
	Investment in research and innovation
Trade and logistics facilitation^a^	Supporting value added logistics activities
	Maintaining high quality and cost-efficient business services (e.g. efficient cargo handling and clearance)
	Integration with maritime supply chains
	Improving ships Just-In-Time (JIT) and virtual arrival
	Supporting JIT import and export
	Optimising port-ship-truck operations (e.g., use of terminal operating system (TOS) for berth planning, and yard and equipment scheduling, planning, and allocation)
	Automation of cranes, including port trucks such as the use of Automated Guided Vehicle (AGV)
	Automation of gates (automated gateway system)
	Using automated mooring systems for ships
	Streamlining the number of containers moves (throughput)
	Improving truck and rail traffic, and inland navigation access
	Facilitating and promoting adequate (multimodal) infrastructure
	Building and integrating dry ports and inland container depots (ICD)
Digitalisation^a^	Use of a single window and port community system to service ships and land transport including other stakeholders (one-stop-shop)
	Employment of paperless business and operations (e.g. electronic data interchange (EDI), E-document program, RFIDs)
	Utilising digital connectivity technologies and data analytics (e.g. Internet of Things (IoT), and big data cloud, and edge computing)
	Utilising blockchains (e.g. Digital Ledger Technology, electronic bill of lading (Bolero))
	Cyber security measures

^a^Measures within these actions improve air quality as well as climate change mitigation due to reduction of energy consumption

### Port sustainability implementation schemes

While important as a foundation for enhancing sustainability, actions and measures—operational and technical—don’t work standing alone and are not a silver bullet. Furthermore, as argued in the introduction, some of the actions and measures are mainly proposed and thus not highly implemented in real world scenarios. It is worth noting that regardless of ports’ geographical,^8^ political, operational, regulatory, financial, and surrounding community settings, which all shape and design sustainability initiatives, ports can offset their environmental issues and sustain social and economic sustainability including maritime supply chains (I2S2 2013; Puig et al. 2014; Acciaro et al. 2014; Asgari et al. 2015; Poulsen et al. 2018; Notteboom et al. 2020). Particularly, the port authorities which manage the landside and seaside operation through four key functions as landlords, regulators, operators and community managers (I2S2 2013; Poulsen et al. 2018), can play influential roles in ecological protection planning and future sustainable development (Yap and Lam 2013). The port authority can be either under the Hanseatic tradition where the local government or municipality have a strong influence in port governance (the port authority has higher autonomy), or Latin tradition where the central government plays a more prominent role (the port authority has less autonomy and public authorities are stronger) (Notteboom and Lam 2018).

Thus, to implement sustainability actions and measures, either port or public authorities, even in cooperation with the private sector, ensure and drive proper implementation, i.e., increase the uptake of actions and measures. It is essential to note the difference between the actions and measures and the implementation scheme as many studies still use them interchangeably. Previous research referred to such schemes as institutional aspects (Laxe et al. 2017), and management and policies (Lam and Notteboom 2014; Bjerkan and Seter 2019). Based on best practices of front-runner ports and literature, the implementation schemes can be categorised into regulations and standards, incentives and disincentives including grants, voluntary and compulsory agreements, and training and information sharing. Port or public authorities utilise these schemes toward port operators and tenants, ships and land transport. While these schemes were mainly utilised for environmental dimensions, they are compatible with other sustainability dimensions (Alamoush et al. 2021b).

### Regulations and standards

Globally, various conventions, standards, and frameworks exist to support sustainable development particularly in implementation of the UN SDGs, in that countries play significant roles in their implementation. The Paris Agreement, under the United Nations Framework Convention on Climate Change and the Sendai Framework for Disaster Risk Reduction 2015–2030, contribute to low-carbon and resilient development for climate change. Moreover, there is a growing United Nations’ concern regarding oceans and coasts which was manifested in 2017 by the declaration of United Nations Decade of Ocean Science for Sustainable Development, 2021–2030. The declaration entails that ocean science will be key in developing effective measures for coastal protection and coastal zone management, as well as climate-risk assessment, adaptation and resilience-building for seaports and other coastal transport infrastructure (UNCTAD 2019a). Against this background, while countries’ political and economic actions boost implementation of such global sustainability efforts, definitely this has implications on port standards and regulations, considering ports as a national identity and under countries’ jurisdiction.

Additionally, other relevant international and national maritime regulations exist, which could be utilised by ports to implement sustainability measures. The international regulations related to maritime transport include the UN Convention on the Law of the Sea (UNCLOS), particularly those articles requiring states to reduce shipping pollution (i.e., 192, 194, 211, and 212), in addition to several IMO conventions (e.g., SOLAS for safety of life at sea,^9^ International Convention for the Prevention of Pollution from Ships (MARPOL)^10^ for the environmental protection, and FAL convention for trade facilitation), Maritime Labour Convention (MLC) for workers’ rights (seafarers), London Convention and Protocol on prevention of marine pollution by dumping of wastes and other matters at sea, and World Trade Organization agreements and provisions, among others (Alamoush et al. 2021a). In line with the Paris Agreement, ongoing IMO work is accelerating towards targets for ships’ GHG emissions reduction (i.e., the IMO Initial GHG strategy (IMO 2018c)). As such many regulations are anticipated to be introduced to curb shipping emissions (e.g., the new existing energy efficiency design index (EEXI), and carbon intensity indicators (CCI)) (Clarksons Research 2020b). Consequently, ports will need to cooperate with IMO and definitely play a regulatory role for the implementation of such targets.

Furthermore, there exist regional regulations, such as those environmental directives and regulations in the EU region and countries, e.g., European Commission (EC) directive No. 2015/757 on monitoring shipping emissions, and EU green deal and climate law. Likewise are national regulations, e.g., Australian Environmental Protection Act, Singapore Environmental Protection and Management Act (Roh et al. 2016), and Hamburg Climate Change Act 2020. Various ports implement environmental management systems and plans (EMS, PERS, EMAS, SDM, ISO 14001, etc.) to maintain national regulatory compliance (Hossain et al. 2020).

In brief, maritime administrations and port authorities have a significant role in policy making (Schröder-Hinrichs et al. 2020). The public and port authorities’ policies and priorities are derived from the aforementioned international and local environmental, social, and economic regulations. Port authorities, including port states, enact regulations and make application of measures (by port operators, ships, and land transport) legally binding through legislation; that is, to minimize ports’ environmental impacts and embed sustainability in operations (Puig et al. 2014; Acciaro et al. 2014). Examples of ports’ regulatory power over shipping pollution is the combat of ship-source pollution and the proliferation of invasive alien species through implementation of the International Convention for the Control and Management of Ships’ Ballast Water and Sediments (2017). Similarly, the IMO regulation for ships’ sulphur cap (entered into force 2020), aims at decreasing sulphur in fuel from 3.50% down to 0.05%. Therefore, under MARPOL responsibility, ports assume a significant role in enforcement, compliance and monitoring of the cap. With regard to efforts toward terminal operators, port authorities may enforce liability standards and require operators to control emissions by, for example, banning and restricting CHE using fossil-fuels (Notteboom and Lam 2018). Additionally, ports may provide guidance documents (what can and cannot be done), and thus guide tenants to comply with regulations (PIANC 2014). The regulation is the ultimate backstop for sustainability and technological measures implementation (Bouman et al. 2017).

### Incentives and disincentives

Incentives*,* or as regularly called, environmentally differentiated port fees, and grants (subsidies), are approved by public or port authorities beyond regulatory requirements. The incentive functions as carrot vis-a-vis the stick of charges (e.g., the environmental pollution charges and extra tariffs) (Lam and Notteboom 2014). Many authorities provide funds for operators and tenants; thus, funding and grants are vital to bear the high costs of technical measures including its operation. Some ports provide ships with incentives for burning cleaner fuel, connecting to OPS, reducing speed (the case of the Port of Long Beach) and slow steaming (Roh et al. 2016). Various indices exist, either led by a ports or by the industry. Indices are used to incentivise ship and port operators who implement safety, security and environmentally friendly measures, e.g., the environmental shipping index (ESI), clean shipping index (CSI), green award (GA), and GHG emission rating (GHG ER), Green Marine, etc. Alternatively, port authorities may modify the tariff and formulate different rates (pricing mechanisms) for ports operators, ships, and land transport (PIANC 2014), in order to pay for the externalities and damages they cause. The extra tariffs on polluters may be used to incentivise those who demonstrate green performance (COGEA 2017). It is worth noting that incentives, based on indices, are not common owing to the onerous efforts for registration, little proportion of rebates against the cost of technology. With regard to disincentives, without uniform application they can compromise port competitiveness (Alamoush et al. 2021b).

### Voluntary and compulsory agreements

With no legal obligations, ports may sign voluntary agreements with polluters, or other social and economic forums/unions, to transform to more sustainable performance. For example, ports may sign voluntary agreement with ships for speed reduction while approaching ports, among others (Gibbs et al. 2014). Volunteering initiatives, including volunteer planning that involves all stakeholders, such as the public, are also one of the ways to advance sustainability without compromising port competitiveness. In a similar fashion, compulsory agreements can be signed with port operators, ships, and land transport, through concession contracts and licences to operate, to include the sustainability actions and measures during their operation within the port (Lam and Notteboom 2014; ITF/OECD 2018; Poulsen et al. 2018). For example, concession terms may include criteria for land use, energy, emissions, water and soil, and biodiversity (PIANC 2014). A number of European port authorities include environmental requirements in terminal concession contracts (Notteboom et al. 2012). Furthermore, land transport environmental measures are also included in contracts with port operators (Gonzalez-Aregall et al. 2018).

### Training and knowledge sharing

Port authorities ensure the outreach of sustainability awareness to their employees, port operators, and even ships and land transport (Acciaro et al. 2014; Gonzalez-Aregall et al. 2018). Thus, ports may develop training courses and seminars that aim at changing trainees' behaviour toward better uptake of sustainability actions—within top-management and the front-end staff. In view of this, ports may encourage employees to use environmentally friendly transport such as carpooling, and public transport (I2S2 2013), provide sustainability training courses and guidelines (Roh et al. 2016) and even improve employees’ ICT skills and competencies to better handle digitalisation and automation (Esser et al. 2020). Training needs to include all the identified actions and measures for relevant employees and stakeholders. Environmental awareness training is used as a benchmark in the ESPO’s EcoPorts survey (ESPO 2019). Furthermore, ports may disseminate sustainability information (Wilmsmeier 2020), and promote the green port concept (e.g., green port seminars) within surrounding communities (Roh et al. 2016). The ports’ sustainable awareness training can create a spillover effect over various supply chain members and port stakeholders. Nonetheless, port authorities can serve as central point of knowledge for sustainability, hence cooperation with research institutes is a catalyst that enables ports to share knowledge and experiences with operators (including other ports), tenants, ships, and land transport (PIANC 2014).

## Linkage of port sustainability actions and measures to the UN SDGs

**Fig. 6:**
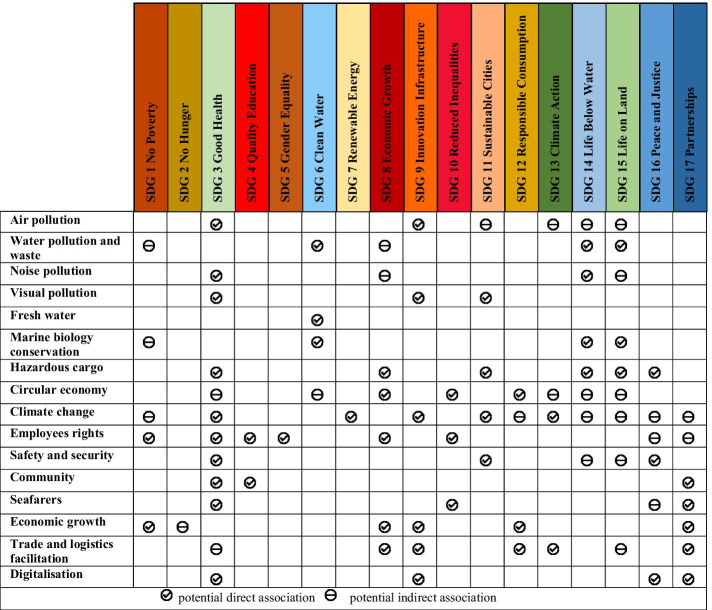
Potential linkage between port sustainability actions (first column) and UN SDGs (first row). *Source*: Authors’ contemplation

Both port sustainability actions and measures, and the sustainable development goals could be seen as catalysts for global sustainability. As has been shown so far, ports can implement actions and measures to improve sustainability while considering the TBLs. While broader in scope, in similar fashion, the UN SDGs address world economic, social and environmental issues, i.e., the TBLs. Then a question may be raised as to whether ports’ sustainability measures and actions can be a foundation and contribute to the UN SDGs. As shown in Fig. 6, and based on matching similarities^11^ between the two, ports’ actions and measures contribute to achieving the UN SDGs, either directly or indirectly, and the contribution is vast. On this basis, the following subsections partially shed light on such contribution.

### Linkage with environmental actions

Port environmental actions can contribute to many SDGs. For example, the air emissions reduction action and measures support Goal 3 (good health), for employees, and surrounding communities,^12^ and protect against ocean acidification (Goal 14 marine life). In addition, lower emissions contribute to Goal 9 (innovation and infrastructure) by using innovative technologies (e.g., electrification, hybridisations, and alternative fuels), and Goal 11 (sustainable cities) considering that ports are integrated with cities. Even port air pollution if not decreased would eventually undermine Goal 15 (life on land—forest and ecosystem). Though most air emissions measures target the reduction of ambient air emissions, they still contribute to reduction of GHG emissions (Goal 13 climate actions).

Water pollution and waste actions and measures are important to maintain a good water quality on land, thereby sustaining Goal 6 (clean water and sanitation). On the other hand, while ports control and minimise shipping (e.g., the waste reception facilities in accordance with IMO MARPOL), and port residue, and waste discharge to seas and oceans; life below water improves (Goal 14). Even health of locals and tourists (swimmers), and economics of coastal population, and wildlife ecosystems are protected by actions to prevent water pollution (e.g., from ballast water, and oil spills), which indicates association with Goal 3 (health), Goal 1 (poverty), and Goal 15 (wildlife), respectively.

Noise pollution actions protect against deterioration of marine life (Goal 14 life underwater), and protect the safety and health of port workers, seafarers, and communities (Goal 3 health and wellbeing). Visual pollution actions such as building colouring and camouflage and planting trees improve the aesthetics of the city, i.e., Goal 9 (innovation and infrastructure), and Goal 11 (sustainable cities and communities). Measures to reduce light pollution improve the health of employees and surrounding people (Goal 3 health). Importantly, sustainable water consumption actions definitely preserve drinking water and thus contribute to Goal 6 (sustainable management of water).

Marine biology conservation actions are various and thus contribute to different goals. For example, limiting sediments and dredging improve marine life, and stop damage to biodiversity (Goal 14). Flood control measures protect habitat quality and flora and fauna (Goal 15 wildlife), and the quality of drinking water (Goal 6). In addition, marine biology measures protect coastal cities’ economies by not harming fishing stocks and tourism, thus fighting poverty (Goal 1). Innovation of the circular economy contributes to many goals. For example, reuse and recycling of materials contributes to Goal 12 (responsible consumption), and Goal 11 (sustainable cities). In addition, reduction of waste in and around ports protects underwater life (Goal 14), and wildlife and the land ecosystem (Goal 15), and also protects the health of communities and tourists (Goal 3 good health).

Considering that the world suffered and still suffers from the consequences of the COVID-19 pandemic, tackling climate change is another eminent global issue (shock) of immediate concern. Ports can take mitigation and adaptation actions in this regard, and hence, they all contribute to Goal 13 (climate actions), and once ports improve infrastructure, they contribute to Goal 9 (innovative infrastructure) and Goal 11 (sustainable cites). Developing efficient hinterland connections and intermodal links has a similar impact. Reduction of GHG emission, for example by electrification and onshore power supply, also protects against ocean acidification and underwater noise (Goal 14). Energy efficiency measures minimise energy consumption (Goal 12 responsible consumption), widen the access to renewable energy such as wind, solar, ocean, and geothermal (Goal 7 clean energy), and improve profitability thus contributing to Goal 8 (economic growth) and Goal 1 (reduction of poverty). Reduction of congestion in and around the port, and improving mobility of cargo and employees are examples that boost city and community sustainability (Goal 11), and improve the health by reducing accidents and ambient air pollutants (Goal 3).

### Linkage with social actions

Port workers and employees, in addition to seafarers, truck drivers, customers and surrounding communities all are impacted by port operations, externalities and administrative decisions. Similar to the environmental dimensions, social actions and measures also significantly contribute to the UN SDGs. Support of local employment and employees’ right to good welfare and health are a foundation for Goal 3 (health) and Goal 8 (good jobs and economic growth), which ultimately reduce poverty (Goal 1). The non-discriminative employment, and gender inclusion contribute to Goal 10 (reduced inequality) and Goal 5 (gender equality), respectively. The safety and security actions and measures protect ships and ports against terrorism, sabotage and armed robbery, and provide safe shelter, thus maintaining peace in cities and societies, i.e., Goal 3 (health and wellbeing), Goal 16 (peace), and Goal 11 (sustainable cities). Collaboration with communities and supply chains, for example with ships and trucking companies and other stakeholders, leverage Goal 17 (partnerships). Community actions contribute to societal health (Goal 3 health), and education (Goal 4 quality education), and city and community sustainability (Goal 11 sustainable cities), while at the same time integrating and creating synergies with academic institutions improves collaboration (Goal 17 partnerships). Paying considerable attention to seafarers’ rights and welfare is considered a catalyst for sustaining their physical and mental health (Goal 3), reducing unequal treatment (Goal 10), and supporting the collaboration with shipping companies (Goal 17 partnerships). Ports’ actions toward CSR undoubtedly create synergies that open the space for better collaboration and partnerships (Goal 17). The same is true when ports share knowledge, expertise and technological innovations. The social programs, e.g., employees’ welfare, education and training not only improve SDGs but also improve environmental sustainability, particularly when the training contains ways to enhance their sustainability adaptation, thereby improving the whole sustainability performance, i.e., Goal 17 partnership for goals implementation.

### Linkage with economic actions

Although economic actions and measures seem to sustain port profitability and enhance regional economy, they are interconnected with other environmental and social dimensions. Ports implement actions toward attracting foreign funds, and public private partnerships (PPPs) (Goal 17 partnership), thus improving infrastructure (Goal 9 innovation and infrastructure), supporting city economy (Goal 8 economic growth and Goal 1 minimising poverty) and contributing to import and export of food and agricultural production in a way that minimises hunger in countries (Goal 2). So, too, does trade and logistics facilitation, i.e., partnerships (Goal 17), economic growth (Goal 8), and responsible consumption and production (Goal 12) through JIT, responsible procurement and inventory, automation and TOS. Such measures yield trade efficiency and minimise delays and congestion and thus reduce air emissions, e.g., atmospheric emissions that influence health (Goal 3), and CO_2_ that stimulate climate change (Goal 13). Digitalisation measures facilitate efficient trade and bring about technological innovations (Goal 17 partnership and Goal 9 innovations) and maintain transparent communication, while addressing cyber security issues thereby contributing to Goal 16 (peace and strong institutions). Digital technologies minimise human interaction: this is very important during pandemics (e.g., COVID-19) and thus protects the health of port related workers (Goal 3 health) and maintains continuous maritime trade, given that four fifths of world trade is seaborne.

## Discussions and conclusions

Port sustainability initiatives are sporadic, local in dimensions, and mostly implemented in large developed countries’ ports. On the other hand, knowledge of port sustainability is accelerating rapidly, but at the same time remains fragmented, making it hard to collectively assess various research. Just as importantly, maritime transport, including ports, is under scrutiny and tightening regulations to maintain sustainable operations, e.g., decarbonisation, climate change adaptation, labour rights, and streamlined operation through digital technologies. A critical literature review method was utilised to explore various academic peer-reviewed literature, and technical reports in order to build a holistic port sustainability framework. The framework includes 16 categorised actions and a non-exhaustive list of measures (138 measures that are either tabulated or explained within the text). Actions are spread over the three dimensions of sustainability, i.e., economic, social, and environmental (TBLs), which include port operations (internally) while at the same time embracing shipping and land transport (trucks) for further sustainability outreach (externally).^13^ Implementation schemes (institutional, policy, and management measures) were identified and aggregated into four groups, which could be used by public and port authorities and in cooperation with the private sector as tools to drive, enforce and increase the uptake of sustainability measures and actions.^14^ While a comprehensive framework to improve port sustainability performance was identified, association of identified actions and measures with the UN SDGs was highlighted, based on mutual similarities.^15^

In comparison with other reviews of port sustainability, e.g., (Asgari et al. 2015; Sislian et al. 2016; Davarzani et al. 2016; Bjerkan and Seter 2019; Lim et al. 2019), this study is broader and more extensive, and reflects a variety of pragmatic and across-the-board actions and measures: i) it included more studies, presented and aggregated more port sustainability actions and measures that are classified in homogeneous categories and subcategories, ii) it expanded the sustainability dimensions (TBLs) to embrace external logistics and the supply chain, iii) it explained how the actions and measures can be implemented by port and public authorities through the implementation schemes—pathways to drive the uptake, and iv) it pointed out the roles that ports can play in advancing the UN SDGs, and, as far as authors are aware, this is the first study in the field that addresses this topic. Therefore, it can be stated that this review creates a firm foundation for advancing knowledge on port sustainability and its development. The findings indicate that there are a variety of actions and measures that enable ports to maintain sustainable performance within and beyond the boundaries of ports (to supply chains). While at the same time, ports can still capture the TBLs and align sustainability actions with the UN SDGs. Thus, this proposes a change in the way we look at port sustainability.

While it is argued that a great focus is exerted only on environmental issues, other dimensions are also important. Environmental actions and measures by far outnumber the economic and social ones. This can be explained by the focus on green port initiatives both in research and practice, e.g., ESPO EcoPort initiative. As most ports are built around cities, and considering that ports generate externalities and economic benefits, this study highlighted the importance of integrating communities and the employees in ports’ social and economic suitability actions. The same is true regarding seafarers, who are commonly a neglected group in this field. The IMO designated seafarers as key workers and announced the world maritime day theme as "Seafarers: at the core of shipping’s future", which is a key step in settling the ongoing crew change crisis. This study suggests that ports have a role to mitigate this issue and need to pay due respect to the two million seafarers who operate the shipping fleet.

Considering that most of the actions and measures categorised in this study are mainly proposed to advance sustainability, their uptake in global ports seems to be far from complete. This is attributed to different barriers, such as costs, knowhow, and the complexity of port businesses (engaging with various stakeholders in land and sea). Nonetheless, the actions and measures identified are still vital in addressing various challenges. Issues ports have faced during the COVID-19 pandemic (e.g., teleworking, social distancing, safety measures, delays, and capacity utilisation issues), and in light of the fact that seaborne trade is expected to pick up again after the pandemic, sustainability actions and measures elucidated in this study will help accommodate the challenges related to COVID-19, while all together facilitating and streamlining trade, and supporting ports’ long-term sustainable recovery. In line with this, digitalisation, internet of things (IoT), and big data platform, decrease human interactions and, also advance paperless trade, and improve data analytics for better decision making and sustainability performance monitoring. Additionally, many sustainability challenges, such as the climate change mitigation measures, can be seen as opportunities to improve efficiency and make some profit, (e.g., through energy efficiency), among other opportunities such as trade growth, job creation and the adoption of technological innovations.

The fusion of the port sustainability dimensions resulted in forming a well-rounded view of actions and measures not only relevant for ports per se, but also ships and land transport (trucks). The inclusion of supply chain members’ responsibility to implement sustainability throughout their business with the ports widened the concept of port sustainability performance as sustainability challenges have no territorial borders. While this recognises ports’ key role in maritime supply chains, and their being essential nodes between the sea and land, sustainability beyond each organisation’s boundaries is rarely achieved (Poulsen et al. 2018). Ports enforcing shipping and land transport to adopt port-imposed measures, other than those that combine with port measures, such as emission reduction and safety measures, is not yet common. This is attributed to shipping being subject to international regulations and land transport being under varying governance, e.g., private or public. Against this backdrop, implementation schemes, such as enacting regulations in accordance with international and national conventions and provisions, incentives and disincentives, voluntary and compulsory agreements, and training and knowledge sharing, would greatly help in mitigating such challenges and thus drive and appeal to shipping and land transport to improve their sustainable performance.

This study addressed the port’s holistic role in sustainability. Though the framework does not provide a set of actions and measures that guarantee success, comprehensive insights are generated, which have managerial implications that are relevant for port policymakers and managers particularly those who are active in sustainability implementation.

While the framework is holistic in nature, other factors may influence adoption of its various measures and actions, i.e., different sustainability measures being incorporated differently in different ports. The reason is that every port is unique in terms of its geographic, political, governance, community, operational, regulatory and economic settings. Freshwater river ports and saltwater ports have distinct habitats, and therefore different measures are applied. Therefore, it is recommended that ports tailor their actions and measures based on their circumstances, considering the particularities of port operation and development undertakings. Examples of different port circumstances are: type of trade (e.g., cruise, break-bulk, general cargo, container, bulk), emission focus (SOx, NOx, PM, OVC, CO_2_), management role (e.g., landlord, operator, hybrid), model of managed business (terminals, industrial activities, ferries, bridges) and geography (fresh or salt water, estuary). A case in point, the Port of Los Angeles’ sustainability actions focus on health risk reduction, air and water quality, energy and climate change, relationships with stakeholders, habitat protection, open space and urban greening (Roh et al. 2016). In the same way, the EU ports have prioritised a different ten environmental actions (ESPO 2019).

It should be born in mind that just as the presented measures and actions are not one-size-fits-all**,** so, too, the implementation schemes are not one-size-fits-all. A balance among all the TBL aspects is essential for achieving long term sustainability. A mix of such institutional and policy instruments (optimal implementation schemes) needs to be considered, based on ports’ national and local circumstances. Nonetheless, ports can configure their role in sustainability in that the implementation schemes would address some sustainability implications. Sustainability implications can be in terms of costs (financing measures), i.e., ports need funds and grants to adopt the measures. The same is true regarding technical (technology transfer), and capacity building (new capabilities development) requirements, particularly in developing countries’ ports. Caution should be exercised to minimise the risk of impacting economic sustainability; in other words, introduction of strict regulation, extra tariff, taxes and charges may repel a port’s customers (chains), and thus the port would lose them to other ports with less strict strategies (port competitiveness issue). Put differently, ports have role to transit the maritime transport toward sustainability, but these efforts should not impede the flow of world trade. Along these lines, and against this background, it should be borne in mind though, that in order to have successful implementation of port sustainability, monitoring of port sustainability performance is vital to establish a baseline. Also, collaboration, cooperation and coordination (the 3Cs) among all stakeholders is important to facilitate sustainability implementation.

The 17 UN SDGs cover a much wider and global spectrum of sustainability objectives; therefore, a novel attempt was made to identify their potential association with the port’s sustainability actions and measures. As manifested, ports’ sustainability actions are associated with all the UN SDGs. Thus, ports can address their sustainability while associating their actions with all the 17 goals, and in so doing, ports can demonstrate their sustainability approaches beyond customary practices. Additionally, exploiting the actions and measure, and aligning them with UN SDGs, ports may prepare sustainability reports and as well create Key Performance Indicators (KPIs) to assess sustainability performance. Similarly, regional port organisations and association may use the whole framework to benchmark ports (comparability).

In terms of *contribution*, the identified state-of-the-art holistic actions and measures, and implementation schemes work as a tool that assists port policymakers and managers, and terminal and logistics operators in different ports regardless of ports’ circumstances. At the heart of this, the study provides firm insights and a decision support system to help ports establish holistic and global sustainability policies, while at the same time paying consideration to the UN SDGs. This enables: gauging and improving the port sustainability performance and implementation, positioning ports as stewards of the environment and society, by eliminating externalities, and creating opportunities to act ahead of forthcoming strict regulations while being resilient against shocks. Academically, one considerable weakness of the literature on sustainability is the assumption that ports sustainability actions are treated as implemented, which might not be the case in many ports. The clarifications of various actions and, importantly, how they can be implemented (implementation schemes) have provided a new perspective in the field of sustainability research. The identified actions and measures enable researchers to have rapid and holistic understanding of port sustainability and thus contribute to academic dialogues and future research cross-pollination.

*Future research* may use the framework to examine and test port sustainability implementation, and the influence of TBLs dimensions on each other. This entails, in addition, validating the actions and measures through survey questionnaire. It is also desirable to consider mapping the level of awareness of port managers and policymakers through case studies and other form of empirical research. Empirical research, based on real experience, supports port policy and decision makers considering that much of the literature is hypothetical and conceptual. While it is still argued that the COVID-19 pandemic has influenced port sustainability, future research may empirically investigate COVID-19 impacts using the identified port sustainability actions and measures. Further research may investigate what ports require to implement the actions and measures in terms of capacity building, motivations, funding and technology transfer, in addition to identification of relevant factors that affect the implementation. Since sustainable port policies, either internally within ports or externally toward shipping, are relatively mature, it is useful to further the investigation toward land transport as this topic is rarely discussed. Ports that implement sustainability measures are not widely addressed in the literature, except for a few ports in North America, Europe and the Far East of Asia, thus, investigation of developing countries’ ports sustainability is an important research agenda.

With respect to *limitations*, the study focused on port side (terminal operators) and, for external port sustainability only on the key maritime supply chains (ships and land transport). Other supply chain members, e.g., stevedoring, freight forwarders, shippers, railways and inland waterways, were not looked into deeply in the analysis. While it appears that actions and measures in addition to the implementation schemes are considered applicable to other members of supply chains, future research may thus include the dynamics of more supply chain members through port external sustainability. Another limitation is that this study is based on critical review, hence, subjectivity of data selected could be argued to pose an issue. Given the progression and the large throughput of port sustainability actions in the literature, the delineation of number of included studies maintained relevant applicable studies. Additionally, attempts were made to check the reliability of all selected studies (see methods) to minimise such limitation.

Categorisation of actions is generally inclusive though actions might be interconnected, meaning that an action under one category may contribute to other categories, and therefore different categorisation (aggregation) may appear in different studies. While the end goal is still similar, i.e., broader port sustainability, this limitation was mitigated by indicating any other contribution to another actions when there is overlap. Although the actions presented address all port sustainability dimensions, it cannot be claimed that a non-exhaustive list of measures was put together to execute these actions, and so different measures can be added for further investigations. Finally, linkage between port actions and measures and the UN SDGs is based on similarities (extracted from the literature) between the end goal of the two, which is subject to the authors’ judgment, and thus other interpretations and linkages may exist. Nonetheless, this opens the space for further investigation of ports’ novel role in contribution to the UN SDGs, preferably through empirical research.

## Data Availability

Not applicable.

## Abbreviations

CHE: Cargo handling equipment
CO_2_: Carbon dioxide
CSR: Corporate social responsibility
EMAS: European Union’s eco-management and audit scheme
EMS: Environmental management systems
ESPO: European Seaport Organisation
GHG: Greenhouse gas
IMO: International Maritime Organisation
NOx: Oxides of nitrogen
OPS: Onshore power supply
PERS: Port environmental review system
PM: Particulate matter
SDM: Self-diagnosis method
SOx: Oxides of sulphur
TBL: Triple bottom line
TOS: Terminal appointment system
UN SDGs: United Nations’ Sustainable Development Goals
VOC: Volatile organic components

## Footnotes

1 https://unctadstat.unctad.org/wds/TableViewer/tableView.aspx?ReportId=194889.

2 Many shippers have joined forces together with some carriers in the clean cargo working group with the objective to better understand and assess the emission footprint of cargo transport (PIANC 2014).

3 Involve transferring traffic, currently carried by truck, onto other modes, such as rail or inland waterways.

4 Presented the port authorities guide for sustainable ports, and identified 13 environmental and sustainability issues related to port operations and their related logistic chains.

5 Studied the global ports environmental initiatives in ten areas.

6 The Intergovernmental Panel on Climate Change (IPCC) predicted, in 2007, that during the twenty-first century, sea level would rise between 18 and 59 cm (PIANC 2014).

7 The IMO and the International Labour Organization regulate the shipping industry, for clean and safer oceans, and social aspects of seafarers, respectively.

8 Either a freshwater river system, estuary or saltwater harbour, thus this defines what environmental issues are encountered and how they are handled (I2S2 2013).

9 Relevant to ports safety and security are the International Ship and Port Facility Security Code (ISPS Code) and the International Safety Management (ISM) Code under IMO SOLAS. The IMO intends to include the cyber security standards for shipping and ports within the ISM code by 2021.

10 The IMO MARPOL deals with the prevention of shipping pollution, i.e., air emissions, oil chemicals and other hazardous substances pollution, ballast water treatment, ship recycling, the use of harmful paint (antifouling).

11 Each SDG has targets that enhance the TBLs of sustainability, which we check and then match the SDG with the previously identified classification of port TBLs sustainability actions. Three researchers participate in this exercise.

12 More than a billion people live in coastal areas and this number is increasing sharply (IMO).

13 This answers RQ1: What are the categories of ports’ sustainable actions and measures to improve overall port sustainability internally in the port side and externally in the sea side (shipping), and in land transport (trucks)?

14 This answers RQ2: How port sustainability measures and actions can be implemented (implementation schemes)?

15 This answers RQ3: What is the linkage between port sustainability measures and the United Nations Sustainable Development Goals (UN SDGs)?
